# The differential statin effect on cytokine production of monocytes or macrophages is mediated by differential geranylgeranylation-dependent Rac1 activation

**DOI:** 10.1038/s41419-019-2109-9

**Published:** 2019-11-21

**Authors:** Hang Fu, Mohamad Alabdullah, Julia Großmann, Florian Spieler, Reem Abdosh, Veronika Lutz, Katrin Kalies, Kai Knöpp, Max Rieckmann, Susanne Koch, Michel Noutsias, Claudia Pilowski, Jochen Dutzmann, Daniel Sedding, Stefan Hüttelmaier, Kazuo Umezawa, Karl Werdan, Harald Loppnow

**Affiliations:** 1 Universitätsklinik und Poliklinik für Innere Medizin III, Universitätsmedizin Halle (Saale), Martin-Luther-Universität Halle-Wittenberg, 06120 Halle (Saale), Germany; 2 Institut für Molekulare Medizin, Universitätsmedizin Halle (Saale), Martin-Luther-Universität Halle-Wittenberg, 06120 Halle (Saale), Germany; 3 Department of Molecular Target Medicine, Aichi Medical University School of Medicine, 480-1195 Nagakute, Aichi Japan; 4 Present Address: Pädiatrische Immunologie, Otto-von-Guericke-Universität Magdeburg, Universitätsklinikum Magdeburg, Leipziger Strasse 44, 39120 Magdeburg, Germany; 5 Present Address: Institut für Molekulare und Klinische Immunologie, Otto-von-Guericke-Universität Magdeburg, Leipziger Strasse 44, 39120 Magdeburg, Germany; 6 Present Address: Zentrum für Tumor- und Immunbiologie (ZTI), Forschungsbereich Gastroenterologie, Philipps-Universität Marburg, Hans-Meerwein-Strasse 3, 35043 Marburg, Germany

**Keywords:** Interleukins, Atherosclerosis

## Abstract

Monocytes and macrophages contribute to pathogenesis of various inflammatory diseases, including auto-inflammatory diseases, cancer, sepsis, or atherosclerosis. They do so by production of cytokines, the central regulators of inflammation. Isoprenylation of small G-proteins is involved in regulation of production of some cytokines. Statins possibly affect isoprenylation-dependent cytokine production of monocytes and macrophages differentially. Thus, we compared statin-dependent cytokine production of lipopolysaccharide (LPS)-stimulated freshly isolated human monocytes and macrophages derived from monocytes by overnight differentiation. Stimulated monocytes readily produced tumor necrosis factor-α, interleukin-6, and interleukin-1β. Statins did not alter cytokine production of LPS-stimulated monocytes. In contrast, monocyte-derived macrophages prepared in the absence of statin lost the capacity to produce cytokines, whereas macrophages prepared in the presence of statin still produced cytokines. The cells expressed indistinguishable nuclear factor-kB activity, suggesting involvement of separate, statin-dependent regulation pathways. The presence of statin was necessary during the differentiation phase of the macrophages, indicating that retainment-of-function rather than costimulation was involved. Reconstitution with mevalonic acid, farnesyl pyrophosphate, or geranylgeranyl pyrophosphate blocked the retainment effect, whereas reconstitution of cholesterol synthesis by squalene did not. Inhibition of geranylgeranylation by GGTI-298, but not inhibition of farnesylation or cholesterol synthesis, mimicked the retainment effect of the statin. Inhibition of Rac1 activation by the Rac1/TIAM1-inhibitor NSC23766 or by Rac1-siRNA (small interfering RNA) blocked the retainment effect. Consistent with this finding, macrophages differentiated in the presence of statin expressed enhanced Rac1-GTP-levels. In line with the above hypothesis that monocytes and macrophages are differentially regulated by statins, the CD14/CD16-, merTK-, CX_3_CR1-, or CD163-expression (M2-macrophage-related) correlated inversely to the cytokine production. Thus, monocytes and macrophages display differential Rac1-geranylgeranylation-dependent functional capacities, that is, statins sway monocytes and macrophages differentially.

## Introduction

Among the central regulators of innate immune responses and inflammation are mononuclear phagocytes, that is, monocytes (Mo) and macrophages (Mac)^1,2^. They are involved in a variety of pathologies related to innate immunity and inflammation, including auto-inflammatory diseases^3^, sepsis^4^, cancer^5^, or atherosclerosis^6,7^. Many, if not all, innate functions of monocytes and macrophages in inflammatory responses are mediated by cytokines^8,9^. In cardiovascular diseases vascular cells may also be a source of cytokines^10–13^, and may be activated, for instance, by interaction with platelets or monocytes/macrophages^14–16^. Along with interleukin-6 (IL-6) or tumor necrosis factor (TNF), IL-1 is a central mediator of innate inflammatory responses^17^.

Besides regulation of cholesterol synthesis, statins also may provide beneficial effects in cardiovascular diseases by regulation of inflammatory responses^18,19^. Both anti-inflammatory^20^ and pro-inflammatory^21–25^ statin effects have been reported. In these papers, freshly isolated monocytes^20^, as well as preincubated cells or cell lines^21–25^, have been used. Besides regulation of cholesterol synthesis, statins interfere with the isoprenylation-pathway^26^, resulting, for example, in regulation of the GTP-activated protein Rac1^27^, which can modulate IL-1β production^28^. Considering the above, we hypothesized that monocytes and macrophages, depending on their differentiation status, may respond differentially to regulation of the isoprenylation pathway, resulting in differential regulation of Rac1 activation and subsequent IL-1 production.

Since the phenotype of the cells used in the literature cited above was not characterized^20–25^, we used various markers to determine the phenotype of the monocytes and macrophages used in the present work. CD14 and CD16 are well-established markers of monocyte subpopulations^29^. CD163 is expressed in macrophages present in atherosclerotic lesions, but is only slightly expressed in monocytes and it is taken as a marker for (anti-inflammatory) M2-macrophages^30–32^. Also, merTK is not potently expressed in monocytes^33^. However, upon monocyte to macrophage differentiation, expression of merTK is upregulated, particularly in M2c-macrophages^34^. Another possible M2-marker is the fractalkine receptor CX_3_CR1^31^. CX_3_CR1^hi^-cells produce enhanced IL-10-levels, whereas CX_3_CR1^low^-cells produce low IL-10-levels, but high IL-6- or TNF-levels^35^. CD86 is an indicator of (pro-inflammatory) M1-macrophages^32,36^. CCR2 (chemokine receptor 2)/CD192 may be helpful for the identification of “M1-monocytic” cells and may indicate inflammatory monocytes^31^. During differentiation of monocytes to macrophages, CCR2 expression is down-regulated^37^. Monocytes and macrophages may produce cytokines to a different degree^38,39^. According to our hypothesis derived above that statin may regulate functions of macrophages and freshly isolated monocytes differentially, we compared statin-mediated innate/inflammatory responses of monocytes and macrophages, characterized by the mentioned surface markers, at the cytokine, isoprenylation, and Rac1 activation level.

We show that cytokine production of freshly isolated monocytes is not altered by statin, whereas the response of overnight-differentiated macrophages is potently altered. Thus, the pleiotropic capacities of statins appear to depend on the differentiation status of the target cell. We propose that the influence of statin on macrophages is not a costimulation with lipopolysaccharide (LPS), but rather a modification of cell differentiation, determined herein as “retainment effect,” which may keep the cells in a “monocyte-like” (activatable) state. In other words, what at first glance looks like a pro-inflammatory statin effect may entail anti-inflammatory consequences by keeping macrophages in a monocyte-like activatable phenotype.

## Results

### Statins retain the cytokine production of monocyte-derived macrophages in the differentiation phase, but do not affect cytokine production of freshly isolated monocytes

**Fig. 1:**
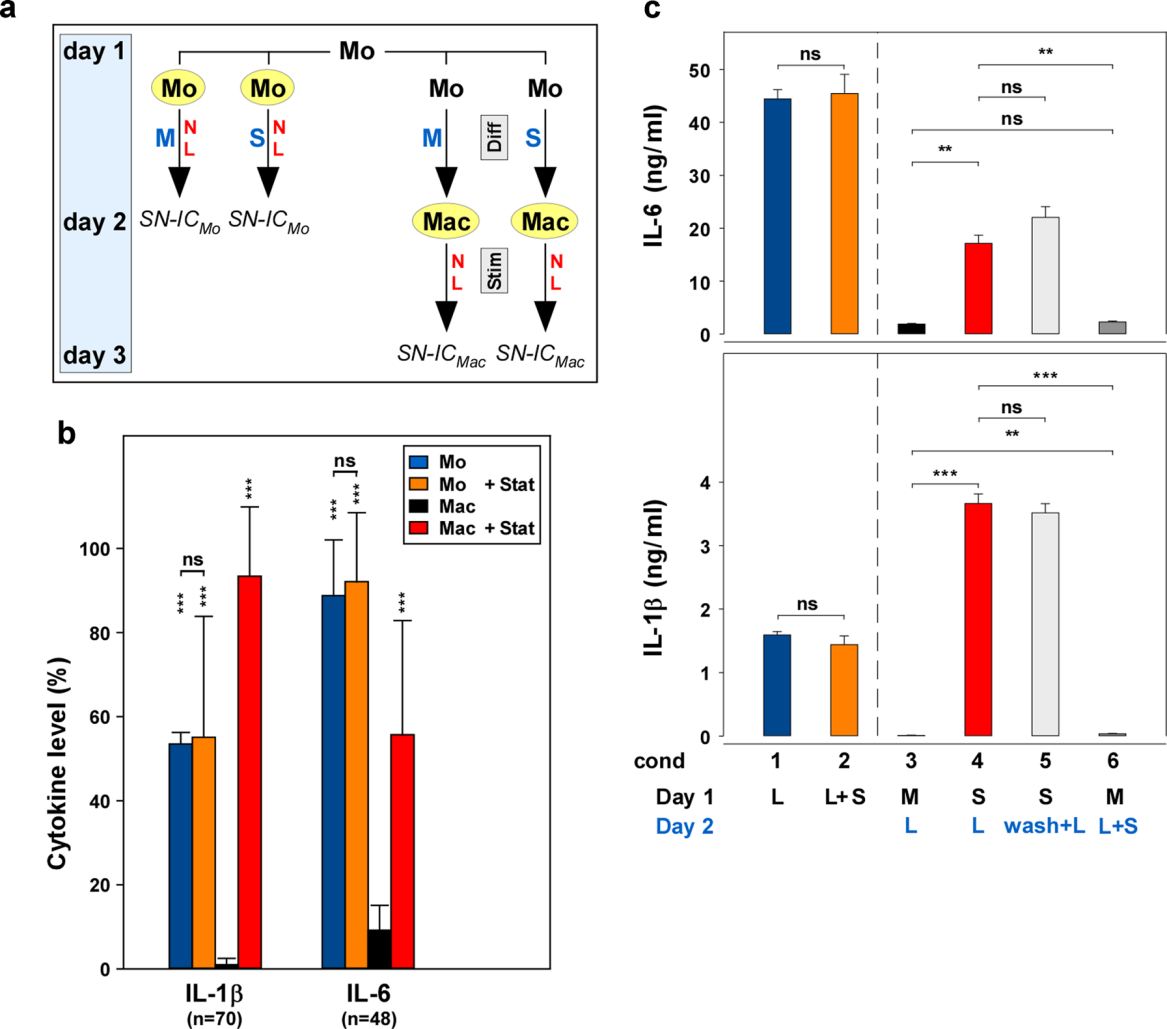
Statins retain the cytokine production of monocyte-derived macrophages in the differentiation phase, but do not affect cytokine production of freshly isolated monocytes. **a** Experimental design. On day 1, mononuclear cells (MNC) were prepared from heparinized whole blood. Monocytes (Mo) were isolated from MNC using CD14-antibodies linked to magnetic beads. Mo (left part of the figure; marked in yellow) were incubated in VLE-RPMI-1640, containing 10% fetal calf serum (FCS), 1% l-glutamine, and 1% antibiotics, without or with statin (10 µg/ml; M or S, respectively; blue letters) and without or with LPS (100 ng/ml; N or L, respectively; red letters), added immediately after the isolation of the Mo. On day 2, the supernatants and/or the cells were harvested and stored for analyses (SN-IC_Mo_). On the other hand, macrophages (Mac; right part of the figure; marked in yellow) were derived from the Mo by incubating Mo without (M) or with statin (S) for 24 h (Diff, differentiation phase; gray box), but without stimulus. On day 2, medium (N) or medium with LPS (L) was added as the stimulus and the cultures were incubated for further 24 h (Stim, stimulation phase; gray box). Thereafter (on day 3), the supernatants and/or the cells of the Mac were harvested and stored for analyses (SN-IC_Mac_). **b** A summary of multiple experiments shows that fluvastatin does not affect the cytokine production of the Mo, but retains the cytokine production of the Mac. Mo and Mac were isolated and prepared as described in Fig. 1a. The cytokine data of the four controls (Mo, Mo + Stat, Mac, and Mac + Stat; blue, orange, black, and red columns, respectively; all LPS-stimulated) always present in the numerous experiments performed in the study (IL-1, *n* = 70; IL-6, *n* = 48) were normalized (%) to the respective highest cytokine level of these four controls. The mean, the SD, and the significance of these data were calculated in SPSS (Levene’s test, Welch’s ANOVA, and Games–Howell post hoc analysis). The asterisks above the columns reflect the significance of “Mac” vs. “Mo”, “Mo + Stat,” or “Mac + Stat”, respectively; other comparisons are indicated by the lines (***p < 0.001; **p < 0.01; *p < 0.05; ns, not significant). **c** The retainment effect of the statin is initiated during the differentiation phase. Freshly isolated Mo (100,000 cells/cm^2^) were incubated in 24-well plates (Nunc) on day 1 and LPS (cond 1) or LPS and fluvastatin (cond 2) were added. Supernatants were harvested on day 2. In order to produce Mac cultures, the Mo were incubated on day 1 in the absence (cond 3) or presence (cond 4) of fluvastatin. On day 2, LPS was added to both cultures (blue letters). In parallel cultures to cond 4 (i.e., statin-pretreated Mac), the statin was removed by a washing step on day 2, before LPS was added (light gray columns; wash + L; cond 5). On the other hand, in parallel cultures to cond 3 (i.e., medium-pretreated Mac), LPS and statin were added simultaneously on day 2 (dark gray columns; L + S; cond 6). Supernatants were harvested after further 24 h. The cytokine concentration was determined in ELISA. Four additional experiments showed similar results. Statistics and color code as in Fig. 1b.

We hypothesized that inflammatory responses of monocytes and macrophages to statins may be different. Thus, we investigated freshly isolated monocytes, as well as macrophages derived thereof by overnight incubation, as outlined in Fig. 1a. A summary of numerous experiments (IL-1β, *n* = 70; IL-6, *n* = 48; Fig. 1b) showed that LPS-stimulated monocytes (blue columns) potently produced IL-1β and IL-6. Fluvastatin did not significantly modulate the cytokine production of these freshly isolated monocytes (orange columns). In parallel, macrophages were differentiated overnight in the absence or presence of statin and were stimulated with LPS not until day 2. In the absence of statin, only little IL-1β or IL-6 production was detectable (black columns). However, in macrophages differentiated in the presence of statin (red columns), the responsiveness to LPS was still present (i.e., “retained”). Further analyses, including mRNA-analyses, biological IL-1-assay^40^, and IL-1β-Western blot, showed the same result (Supplementary Fig. 1a–f). The measurement of caspase-1, the IL-1β-activating enzyme, was in line with a previous report^38^, showing that macrophages expressed less caspase-1 p10 than monocytes (compare Supplementary Fig. 1F; p10 at cond 1 (condition 1) and cond 3).

The above data indicated that preincubation with statin has evoked a retainment of monocyte functions in the macrophages. In order to prove this hypothesis, we designed a washing experiment. In this experiment (Fig. 1c), statin was removed from the macrophages after the differentiation phase (cond 5), before the addition of LPS (light gray column), and another condition (cond 6; dark gray column), where the statin was first added to the macrophages after the differentiation phase, together with the LPS, on day 2. The data show that upon removal of the statin after the differentiation phase, the retainment effect of the statin was still present (cond 5). In contrast, in order to obtain the retainment effect, it was not sufficient to add the statin during the stimulation phase, together with the LPS on day 2 (cond 6). These data indicate that the statin initiates the retainment effect during the differentiation phase, rather than performing a costimulation with LPS during the stimulation phase, as believed previously. The retainment was detectable within 3 to 6 h (Supplementary Fig. 1G).

### Reconstitution and inhibition experiments indicate a role of geranylgeranylation in the retainment of the cytokine production

**Fig. 2:**
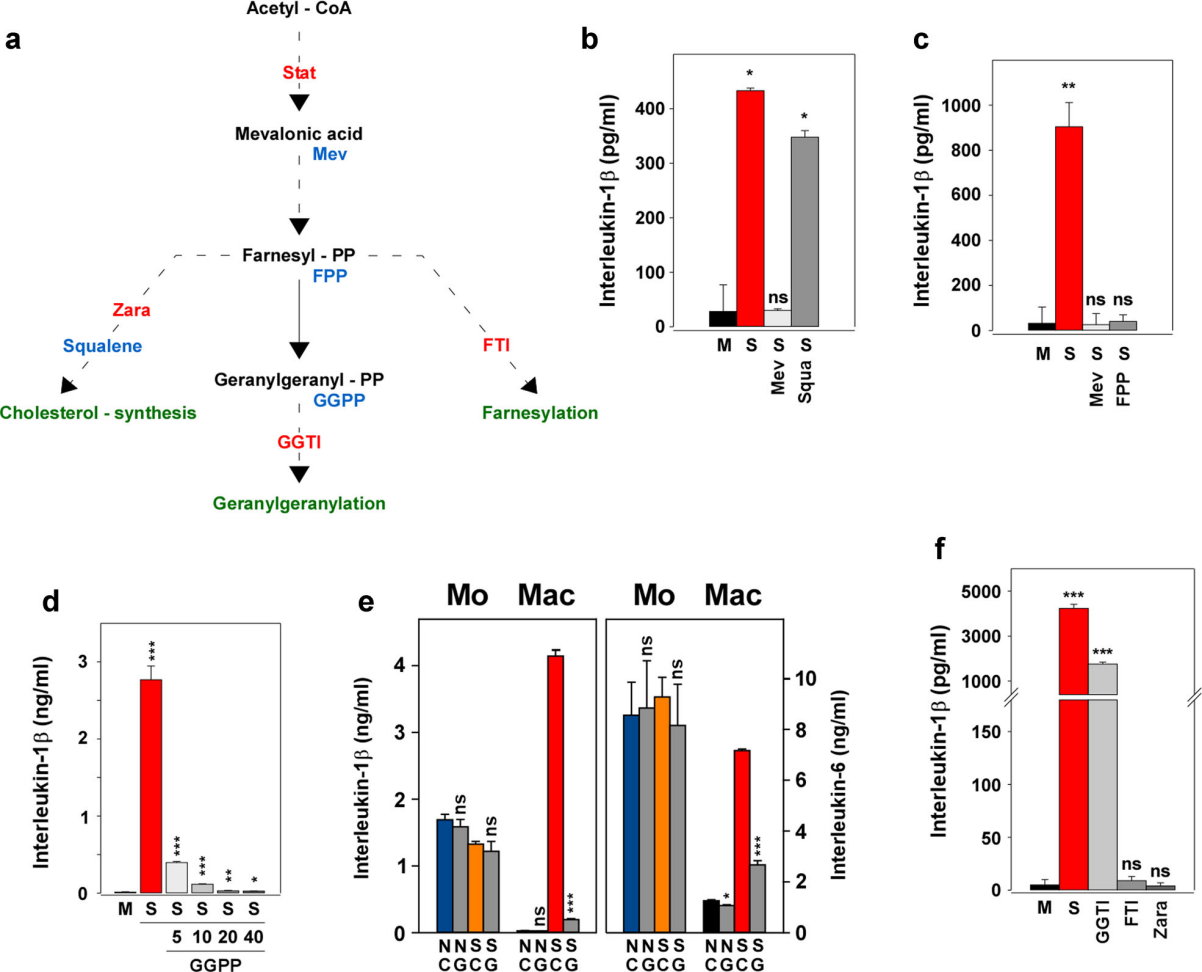
The isoprenoid pathway, rather than cholesterol synthesis, is involved in the retainment effect. **a** Schematic overview of the cholesterol and isoprenoid pathways, as well as the used inhibitors and reconstituting compounds. Red letters, the used inhibitors. Blue letters, the compounds used for reconstitution/spiking. Green letters, the respective pathway. The broken lines indicate omission of steps. FPP, farnesyl pyrophosphate; FTI, farnesyl transferase inhibitor; GGPP, geranylgeranyl pyrophosphate; GGTI, geranylgeranyl transferase inhibitor; Mev, mevalonic acid; Stat, statin; Zara, zaragozic acid A. **b** Mevalonic acid, but not squalene, reverses the retainment effect. Mac (50,000 cells/cm^2^; 24-well plate) were incubated as outlined in Fig. 1a. The cells were preincubated during day 1 with medium (M; black column), statin (S; red column), statin plus mevalonic acid (S Mev; light gray column; 10 µM; Sigma-Aldrich) or statin plus squalene (S Squa; dark gray column; 20 µM; Sigma-Aldrich). On day 2, LPS was added to all cultures and the supernatants were harvested 24 h later. Two experiments with similar results were performed. Data analysis and color code as in Fig. 1c (“M” vs. “S”, “S Mev” or “S Squal”, respectively). **c** FPP, like mevalonic acid, reverses the retainment effect. Experimental design as in Fig. 2b, except for the use of farnesyl pyrophosphate (S FPP, 20 µM; Echelon Biosciences, Mobitec, Göttingen, Germany). Two experiments with similar results were performed. Data analysis and color code as in Fig. 1c (“M” vs. “S”, “S Mev” or “S FPP”, respectively). **d** GGPP blocks the retainment effect in a concentration-dependent fashion. Experimental design as in Fig. 2b, except the use of 5, 10, 20 or 40 µM geranylgeranyl pyrophosphate (GGPP; Echelon). Five additional experiments with similar results were performed. Data analysis and color code as in Fig. 1c (“M” vs. “S” or “S GGPP”, respectively). All comparisons of “S” vs. “S GGPP” were <0.001. **e** GGPP interferes with Mac but not with Mo. Mo and Mac (50,000/cm^2^; 24-well plate) were prepared and incubated as described in Fig. 1a. Statin and GGPP were added on day 1 to Mo and Mac. To Mo LPS was also added on day 1 and the cells were incubated for 24 h. To Mac LPS was added only on day 2 and the cells were then incubated for further 24 h. Color code and statistics (No GGPP vs. With GGPP) as described in Fig. 1c. N, no statin; S, statin (20 µg/ml); C, no GGPP; G, GGPP (5 µM). **f** Like statin, GGTI, but not FTI or Zara, retains the cytokine production of Mac. Mac (100,000 cells/cm^2^; 24-well plate) were prepared in the presence of medium (M) or medium containing fluvastatin (S), geranylgeranyl transferase inhibitor 298 (GGTI; 8 µM), farnesyl transferase inhibitor 277 (FTI; 8 µM) or the cholesterol synthesis inhibitor zaragozic acid A (Zara; 20 µM; Sigma-Aldrich), respectively, for 24 h. On day 2 LPS was added, supernatants were harvested on day 3 and cytokines were measured in ELISA. Two experiments with similar results were performed. Data analysis and color code as in Fig. 1c (“M” vs. “S”, “GGTI”, “FTI” or “Zara”, respectively). Please note the axis break.

In order to investigate the role of the isoprenoid pathway in the retainment effect brought about by the statin(s), we performed reconstitution and inhibition experiments outlined in Fig. 2a. Reconstitution with mevalonic acid (S Mev; Fig. 2b) blocked the retainment effect present in the statin-treated macrophages (S). In contrast, squalene (S Squa), which is a component of the cholesterol synthesis pathway, did not reverse the retainment effect, suggesting that isoprenylation, rather than the cholesterol synthesis pathway, is involved in the retainment effect. Like mevalonic acid, farnesyl pyrophosphate (FPP; Fig. 2c) and geranylgeranyl pyrophosphate (GGPP; Fig. 2d) also blocked the retainment effect. In contrast, in freshly isolated monocytes the addition of GGPP showed no influence on the cytokine production, whatsoever (Fig. 2e). Next, inhibitors of the FPP- or GGPP-transferases were applied (FTI or GGTI, respectively). If isoprenylation would be involved, FTI and/or GGTI were expected to mimic the statin by showing the retainment effect. In line with the above results, inhibition of the squalene synthesis by zaragozic acid A, did not provide the retainment effect, neither did FTI (Fig. 2f). However, blockade of the geranylgeranyl transferase by GGTI provided the retainment effect. These data suggested that geranylgeranylation, rather than farnesylation or cholesterol synthesis, is involved in the retainment effect.

### The blockade of the statin-mediated retainment effect by GGPP is revoked by the addition of the geranylger–anylation inhibitor GGTI

**Fig. 3:**
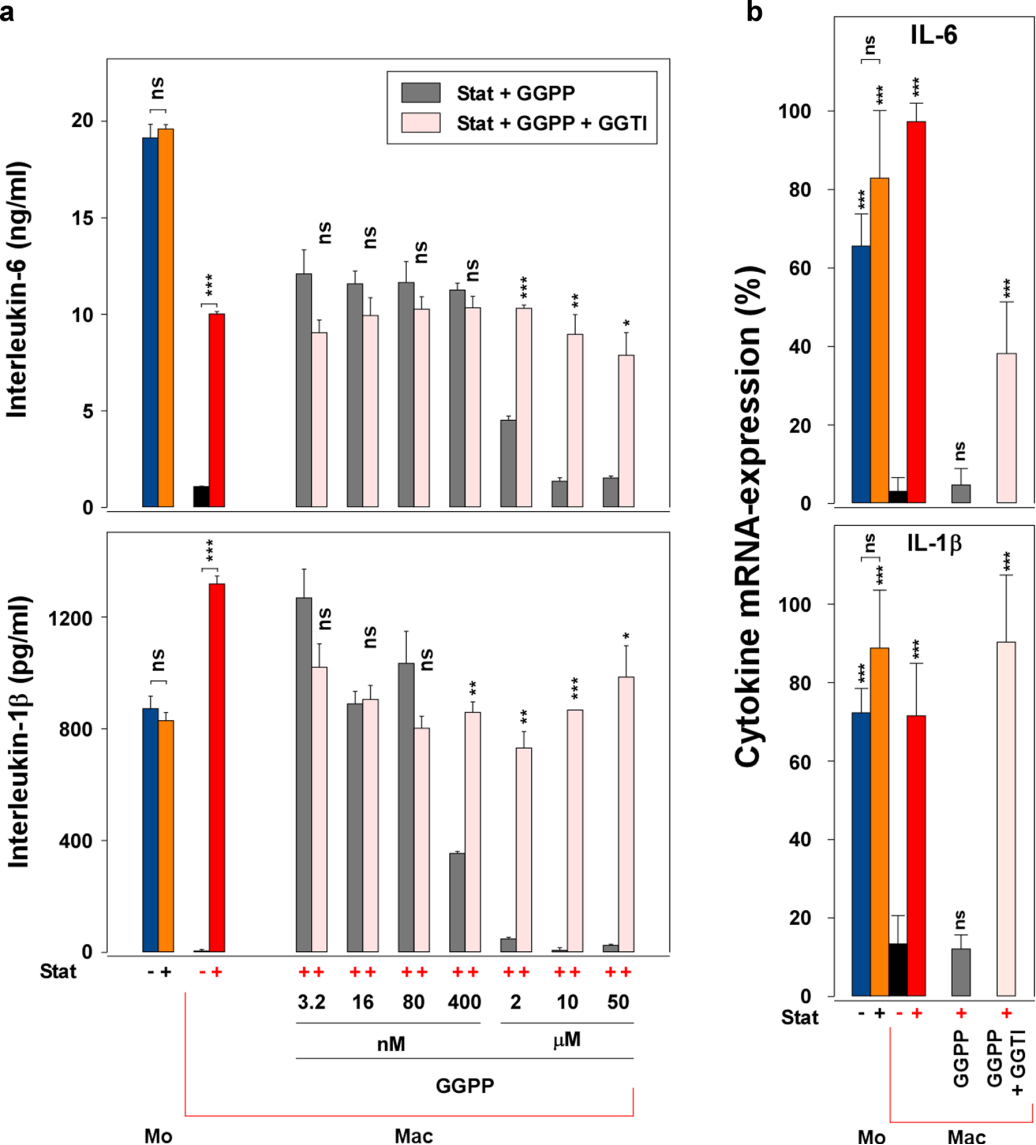
The blockade of the statin-mediated retainment effect by GGPP is reversed by GGTI. **a** GGTI reverses the GGPP effect. Monocytes and macrophages (50,000 cells/cm^2^; 24-well plate) were cultured and stimulated as described in Fig. 1a. The cytokine production of the four controls is presented in the left part of the figure. To parallel cultures of the statin-treated Mac (red column), GGPP at various concentrations, in the absence (gray columns) or presence (rose columns) of GGTI (8 µM), was added on day 1. To all macrophage cultures, LPS was added on day 2 and the supernatants were harvested 24 h later. The cytokine levels were measured in ELISA. One additional experiment with similar results was performed. Data analysis (“Stat + GGPP” vs. “Stat + GGPP + GGTI”; other comparisons are indicated by the lines) and color code (except rose and gray, compare above) as in Fig. 1c. **b** The blockade of the retainment by GGPP and its reversal by GGTI is also detectable at the RNA-level. Monocytes and macrophages were prepared as described in Fig. 3a in 25 cm^2^ culture flasks (Falcon, Corning GmbH, Kaiserslautern, Germany; 100,000 cells/cm^2^; Stat, 10 µg/ml; GGPP, 5 µM; GGTI, 8 µM). IL-1 and IL-6-RNA was measured using the “iScript protocol”. Cytokine levels were normalized to GAPDH. The highest normalized value of each experiment was determined 100% and the mean ± SD of three experiments calculated. Data analysis was performed in SPSS (ANOVA and LSD post hoc; “Mac” vs. “Mo”, “Mo + Stat”, “Mac + Stat”, “Mac + Stat + GGPP” or “Mac + Stat + GGPP + GGTI”, respectively; other comparisons are indicated by the lines; compare Fig. 1c). Color code as in Fig. 3a.

The above experiments indicate a role of geranylgeranylation in the retainment. Thus, we hypothesized that high GGPP-levels or high geranylgeranylation activity, which are probably present in macrophages not treated with statin, reduce the cytokine production. In order to analyze this hypothesis, we added defined concentrations of GGPP to statin-treated macrophages. As proposed, in the cultures containing exogenous GGPP, the cytokine production was reduced depending on the GGPP-concentration (Fig. 3a; gray columns; compare also Fig. 2d). Inhibition of GGPP-transfer by GGTI reversed the blockade of cytokine production caused by the exogenous GGPP (Stat + GGPP), as shown by the rose columns (Stat + GGPP + GGTI). The GGTI recovered the cytokine production even at the highest GGPP-concentration (50 µM). These data support the suggestion that in macrophages differentiated in the presence of statin, the low GGPP-levels caused by the statin may be responsible for the retained cytokine production. Thus, GGPP-levels are probably high in macrophages prepared in the absence of statin, possibly resulting in a phenotypic change (compare below—fluorescence-activated cell sorting (FACS)-data), which is not present in the “statin-treated low-GGPP” cells. The recovery from the blockade caused by GGPP was also observed at the RNA-level (Fig. 3b).

### Rac1 is involved in the retainment of the cytokine production

**Fig. 4:**
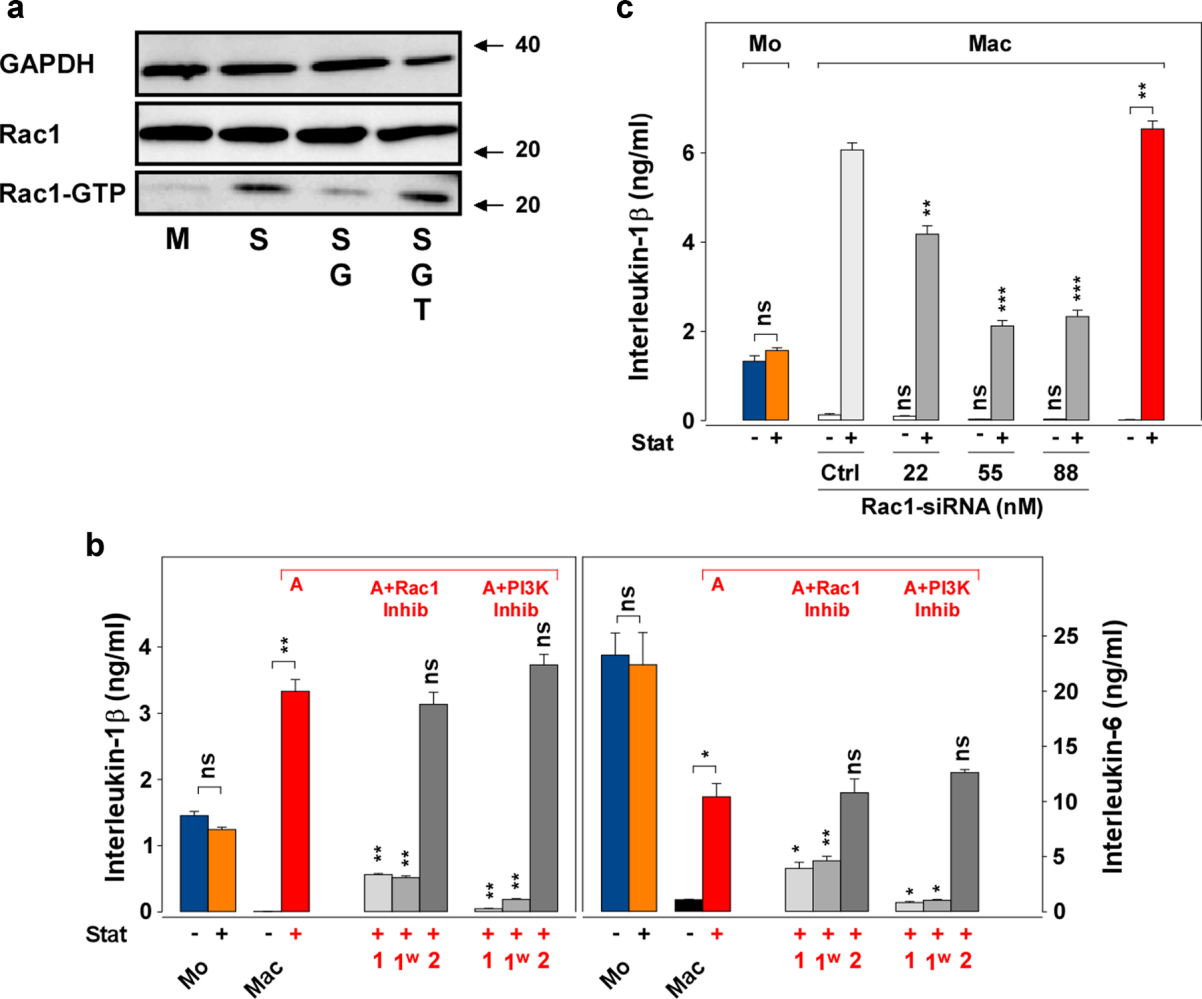
Rac1 is involved in the retainment of the cytokine production. **a** Statin-pretreated Mac (“low-GGPP Mac”) express enhanced Rac1-activation. Macrophages (100,000/cm^2^) were prepared by incubation (75 cm^2^ culture flask; 24 h) in the absence (M) or presence of statin (S; 10 µg/ml), statin and GGPP (SG; G, 5 µM) or statin, GGPP, and GGTI (SGT; T, 8 µM). The cells were harvested and lysed in pull-down lysis buffer. Pull-down for Rac1-GTP was performed with PAK-PBD beads, the beads were washed and used in Western blot. Control samples for GAPDH and total Rac1 (Rac1) were directly taken from the lysate and applied to the Western blot. The numbers at the right indicate the molecular weight (kDa) taken from a molecular weight standard. Four additional experiments with similar results were performed. **b** Rac1- and PI3K-inhibitors block the retainment effect during the differentiation phase. Monocytes and macrophages (50,000 cells/cm^2^; 24-well plate) were incubated as described in Fig. 1a. Parallel to macrophages treated with statin (A; red letter above the columns), macrophages were treated with statin and a Rac1/TIAM1-inhibitor (A + Rac1 Inhib; NSC23766, Tocris, Bio-Techne GmbH, Wiesbaden, Germany) or a PI3K-inhibitor (A + PI3K Inhib; LY294002, Tocris), respectively, both at 10 µM, under the following protocols: “1”, statin and inhibitors were present during day 1 and 2; “1^w^”, statin and inhibitors were present during day 1 and removed by washing on day 2; “2”, statin was added on day 1 and the inhibitors were added on day 2. To all macrophage cultures LPS was added on day 2. IL-1 and IL-6 were measured in ELISA. One additional experiment with similar results was performed. Data analysis (“Mac + Stat” vs. “Mac + Stat + Inhibitors”; other comparisons are indicated by the lines) and color code as in Fig. 1c. **c** Rac1-siRNA blocks the retainment in macrophages. Monocytes and macrophages were cultured as described in Fig. 1a in 6-well plates (100,000 cells/cm^2^; 100 ng/ml LPS), in the absence or presence of statin (10 µg/ml). The control siRNA (Ctrl; 88 nM) or the Rac1-siRNA (both are presented as gray columns; both are “Silencer®select” siRNAs, Ambion) were also added on day 1 in the depicted concentrations to the Mac. LPS was added on day 2 to all Mac and the supernatants were harvested on day 3. IL-1 was measured in ELISA. Two experiments with similar results were performed. Data analysis (“Ctrl” vs. “Rac1-siRNA”) and color code as in Fig. 1c.

A number of cell functions in cell growth or inflammation are influenced by small Ras-superfamily proteins, such as Rac1. For example, the absence of Rac1-activation in THP-1-cells cultured without statin was associated with down-regulated cytokine expression^28,41^. Thus, we analyzed the activation of Rac1, measured as Rac1-GTP, in the retainment model. The Rac1 pull-down assay presented in Fig. 4a shows that untreated macrophages (M) indeed expressed much lower levels of Rac1-GTP than the statin-treated macrophages (S). In line with the above suggestion of a potent role of GGPP, the addition of GGPP to the statin-treated macrophages (SG) resulted in reduced levels of active Rac1-GTP and the GGTI (SGT) reversed this blockade. Supplementary Fig. 2A provides the density analysis of this and three additional blots.

We have shown above that the presence of statin is required throughout the differentiation phase (1st day) of the macrophage culture. If Rac1 or its downstream effector phosphoinositide 3 kinase (PI3K)^42^ are involved in the retainment effect, a similar dependency should be detected. The IL-1 and IL-6 production in statin-treated macrophages was potently inhibited in the presence of the inhibitors throughout day 1 (Fig. 4b; gray columns; “1” and “1w”). However, if the cells were incubated without inhibitor during the first day and the inhibitors were added on the second day (Fig. 4b; “2”), no such inhibition was detectable. These data reinforce that Rac1 and PI3K, in contrast to NF-κB (Supplementary Fig. 2B), are involved in the signaling of the retainment effect during the differentiation phase. The Rac1- and PI3k-inhibitors, and also a p38-inhibitor, blocked the retainment depending on the inhibitor concentration (Supplementary Fig. 2C).

The above paragraphs suggested a potent role of Rac1 in the retainment effect. The GTPase RhoA was activated in parallel to Rac1 (Supplementary Fig. 2D). In order to further analyze the role of Rac1, we performed gene-silencing experiments using Rac1-siRNAs (small interfering RNAs). Figure 4c shows that three concentrations of Rac1-siRNA (dark gray columns) down-regulated the retained cytokine production, as compared to the control siRNA (Ctrl; 88 nM; light gray column). These data further support a potent contribution of Rac1 in the retainment.

### MicroRNAs related to the NF-κB pathway are involved in the retainment

**Fig. 5:**
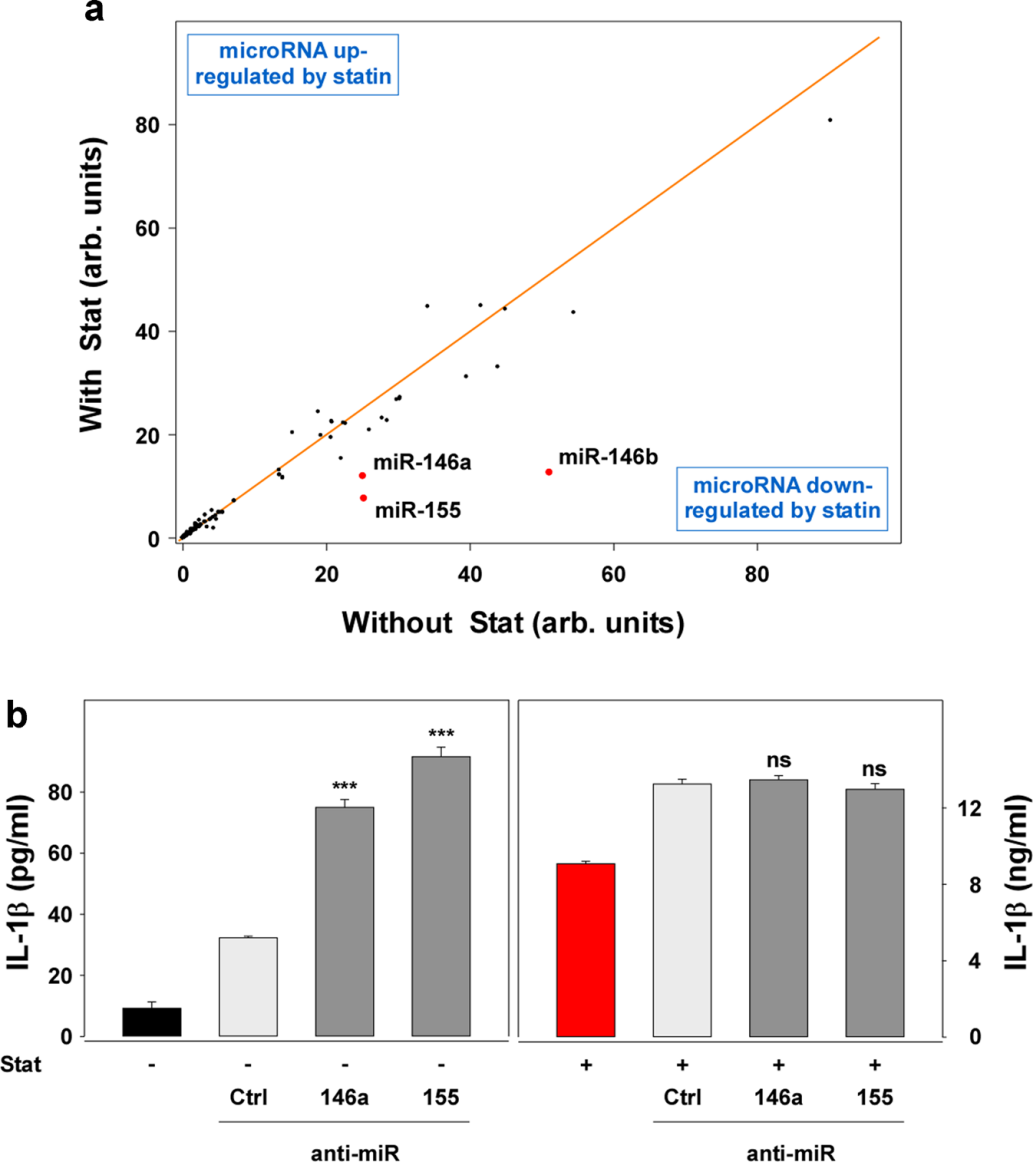
microRNAs 146a, 146b, and 155 are down-regulated in statin-treated Mac. **a** microRNA array. Total RNA was isolated from macrophages differentiated in the absence or presence of statin (25 cm^2^ flasks, 100,000 cells/cm^2^) with the “RNeasy Plus Mini Kit”. Deep sequencing was performed by the “Core Unit DNA”, Universität Leipzig, using the “TruSeq™Small RNA sample prepkit v2” (illumina, San Diego, USA). The mean of the normalized data of macrophages pretreated without statin and macrophages pretreated with statin was calculated and data of samples with a mean >100 counts (192 samples; compare Supplementary Table 2) were included into the analysis and blotted against each other. The orange line indicates unchanged expression. The red dots mark three selected miRs, which were down-regulated in statin-pretreated macrophages, as compared to Mac prepared in the absence of statin (compare Supplementary Table 2). A second array showed a similar result. **b** Blockade of miR-146a and miR-155 reverses the hypo-responsiveness in macrophages only to some degree. Macrophages were incubated as described in Fig. 1a (6-well plate; 100,000 cells/cm^2^). The respective “miRCURY LNA™” anti-miR (Exiqon, Qiagen, Vedbaek, Denmark) were prepared in “Lipofectamine RNAiMax” and 250 µl of this solution was added to 2750 µl of culture medium in the absence (−) or presence (+) of statin. After 24 h LPS was added. After further 24 h, the supernatants were harvested and analyzed in ELISA. Four experiments with similar results were performed. Data analysis and color code as in Fig. 1c (“Ctrl” vs. “anti-miR”).

The Rac1-pathway is probably influenced by microRNAs (miR). Thus, we analyzed the expression of miR in the retainment model. Out of 4445 analyzed miR, 192 miR were differentially expressed in LPS-stimulated macrophages pretreated without statin, as compared to LPS-stimulated macrophages pretreated with statin (Supplementary Table 2). Of these, 100 miR were less expressed in the statin-pretreated macrophages (Supplementary Table 2-(A)) than in the untreated macrophages (Supplementary Table 2B; as indicated by the negative Lg2_A/B_). On the other hand, 92 miR were upregulated (positive Lg2_A/B_). Among the five most potently down-regulated microRNAs were miR-146-a, miR-146-b, and miR-155 (Fig. 5a; red points). The expression of the miR-146a-level in statin-pretreated macrophages was also analyzed by quantitative PCR (qPCR) (Supplementary Fig. 3A; red vs. black column), and, as in the array analysis, was reduced. The three mentioned microRNAs have targets, such as IRAK1 or TRAF6, which are involved in NF-κB regulation, the latter contributing to regulation of cytokine production. Thus, we investigated the role of NF-κB in the retainment using the NF-κB-inhibitor DHMEQ^43^ (Supplementary Fig. 3B). The inhibitor expectedly blocked the IL-1 production of the monocytes. It also blocked the retained IL-1β production in the statin-treated macrophages, supporting the NF-κB dependency of the IL-1 production in the retainment. The blockade by DHMEQ showed the same half-maximal inhibitory capacity in Mo, Mo + Stat, and Mac + Stat, indicating little influence of statin on the NF-kB-activity in the present cells. Analysis of NF-kB-activity using the TransAM assay also showed no influence of the statin on the NF-kB-activity (Supplementary Fig. 2B). The analysis of the NF-kB-, I-kB-, and p38-expression in Western blot pointed in the similar direction (data not shown). It showed a slightly reduced expression of the three factors in the macrophages; however, it did not show an effect of statin or GGPP on the expression of these factors. The addition of anti-miR-146a or anti-miR-155 to the macrophages pretreated without statin increased the cytokine production of the cells (Fig. 5b; left panel), although not to the level of the statin-pretreated cells. In the statin-pretreated macrophages (Fig. 5b; right panel), we observed no significant influence of the anti-miRs, possibly due to the maximal level of the cytokine response present in these cultures. The data indicate that miR-146a and miR-155 may be involved in the retainment, however, to a low degree, and probably synchronous involvement of additional miR is also required. Like these microRNA data, the NF-kB-Western blot discussed above also suggests the involvement of further pathways in the “fine tuning” of the retainment. Rac1, presented in this paper, and also additional components, such as GEF (guanine nucleotide exchange factors), ROS (reactive oxygen species), ubiquination or Ca^++^-levels, may be involved.

### The retainment effect is paralleled by reciprocal expression of macrophage-related surface markers

The above data suggest a retainment of inflammatory functions in macrophages, caused by the statin. Macrophages not receiving the statin treatment, which do not show IL-1 production, may reflect an anti-inflammatory M2-related phenotype, possibly producing IL-10. In line with this suggestion, macrophages cultured without statin treatment showed an increased level of IL-10, as compared to Mo, Mo + Stat, and Mac + Stat (Supplementary Fig. 4A).

**Fig. 6:**
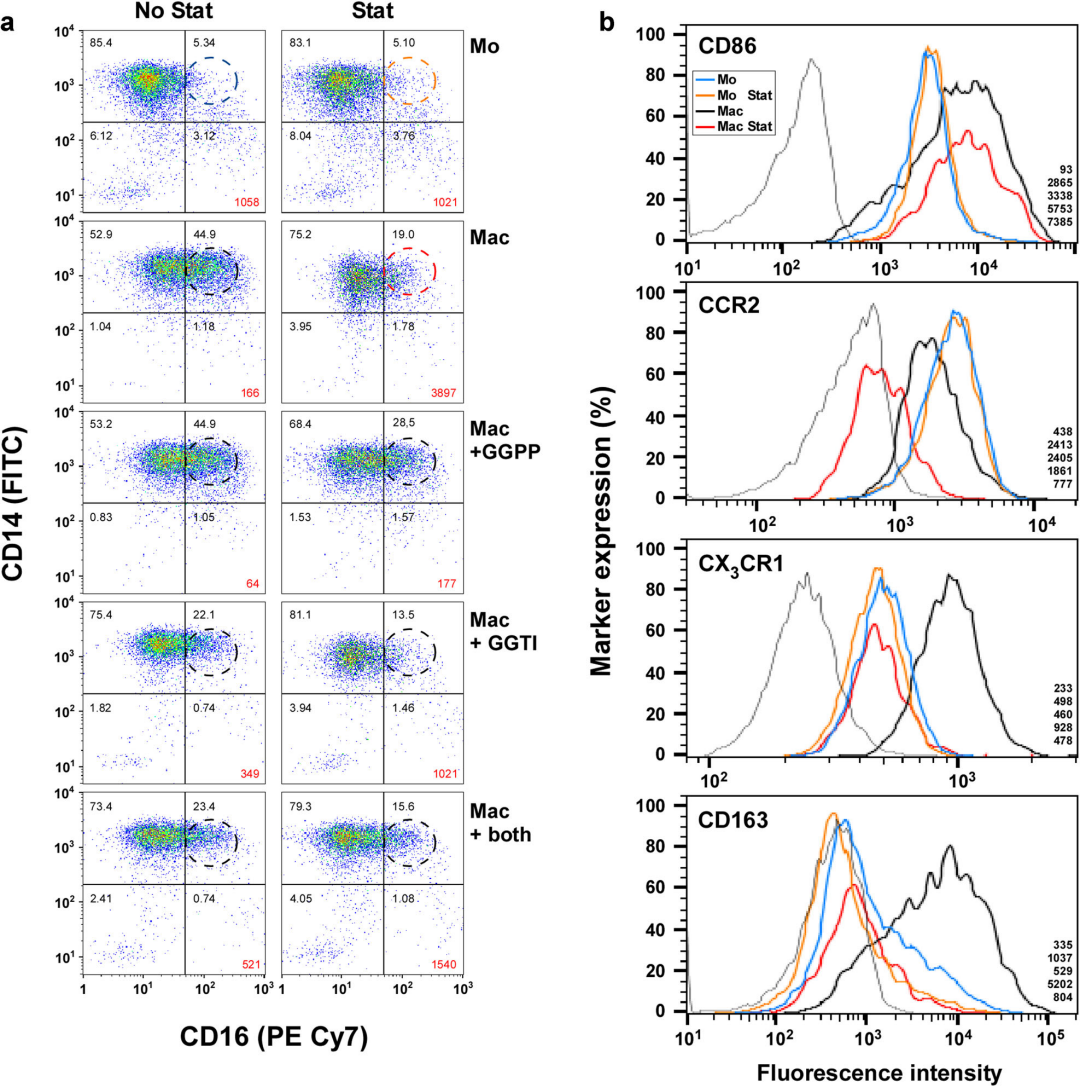
The retainment effect is paralleled by reciprocal expression of macrophage-related surface markers. **a** The enhanced CD14/CD16-expression in macrophages prepared without statin is not observed in macrophages prepared in the presence of statin. The surface marker expression of LPS-stimulated Mo and Mac prepared as described in Fig. 1a was analyzed by flow cytometry. For this purpose the cells (25 cm^2^; 100,000/cm^2^) were harvested, centrifuged (300 × *g*; 10 min), resuspended in 100 µl PBS and transferred into conical 96-well plates (Greiner). The plates were centrifuged (400 × *g*; 3 min) and incubated in PBS containing Zombie Aqua™ (BioLegend, San Diego, USA) for 15 min, followed by centrifugation and resuspension in PBS containing 1% BSA, 0.1% sodium azide and 1 mM EDTA (FACS-buffer). In order to avoid unspecific binding of the antibodies, the cells were incubated with 10% FcR-blocking reagent (Miltenyi) for 15 min at 4 °C in FACS-buffer. Antibodies against CD14 or CD16 (compare Supplementary Table 1) were added (15 min; 4 °C; in the dark). Analysis was performed in a LSR-Fortessa™, using the “FlowJo LLC” software (Ashland, OR, USA). Aggregated cells were excluded by FSC-H- and FSC-A-scatter and dead cells were excluded by gating Zombie Aqua™-negative cells (compare “gating strategy” in Supplementary Fig. 5A–D). The dashed circles indicate the same position of CD14^+^/CD16^+^-cells in each graph. Five experiments with similar results were performed. The numbers in the gates reflect the respective percentages. **B** The CD163- and CX_3_CR1-expression is upregulated in untreated macrophages, but not in statin-treated macrophages. Mo and Mac were prepared as described in Fig. 1a in 25 cm^2^ flasks (57,353 cells/cm^2^). After the respective incubation, the cultures were gently scraped, the cells centrifuged (300 × *g*; 10 min), the supernatants harvested and the cell pellets resuspended twice in 1 ml MACS-buffer (PBS, 2 mM EDTA, 2% FCS; 4 °C). FcR-blocking reagent (2%; Miltenyi) was added for 10 min. Antibodies against CD86, CCR2/CD192, CX_3_CR1 or CD163, or the respective isotype controls (compare Supplementary Table 1; thin gray line in the figure) were added. After 20 min of incubation in the dark, the cells were washed with MACS-buffer and analyzed using a LSR-Fortessa™ (BD Biosciences). Aggregated cells were identified in the FSC-H (forward scatter-high) FSC-A (forward scatter-area) window and excluded from the analysis (compare “gating strategy” in Supplementary Fig. 5A–D). The monocyte region was then determined and gated based on the FSC and SSC (side scatter) parameters. Dead cells were excluded by gating of cells, which were not stained for 7-AAD (7-aminoactinomycin D; BD Biosciences). Visualization and analysis were performed using the “FlowJo LLC” software and the expression of the respective marker (normalized to ratio) was presented. A representative experiment out of seven is shown. The numbers in the lower right corner reflect the MFI (geometric mean) of “isotype control”, “Mo”, “Mo + Stat”, “Mac,” and “Mac + Stat”, respectively, taken from “FlowJo LLC”.

In order to analyze the correlation of the retainment and the macrophage phenotype, we investigated the CD14^−^, CD16^−^, CD64^−^, CD86^−^, CD163^−^, CCR2/CD192^−^, CX_3_CR1^−^, and merTK-expression in FACS-analysis (Fig. 6; Supplementary Fig. 4B, C). Cultured monocytes expressed the same CD14/CD16-level (Fig. 6a) as freshly isolated monocytes ex vivo (data not shown). The distribution of the monocyte subpopulations, as determined by their CD14- and CD16-expression, basically followed the literature^29^. Similar to the finding observed in cytokine production, the statin also did not markedly change the percentage of the classical CD14^+^/CD16^−^-monocytes (85.4 vs. 83.1%). As expected, the CD14^+^/CD16^−^-level in the macrophages was lower (52.9%). Statin reversed the expression almost to the level of the monocytes (from 52.9 to 75.2%). The level of the double-positive cells (CD14^+^/CD16^+^; “intermediate”; dotted circles) in the monocyte populations was also not altered by statin (5.3 vs. 5.1%). However, macrophages contained a much higher level of the intermediate subpopulation (44.9%). As in CD14^+^/CD16^−^-expression, a potent (although not completely to 5.1%) reversal of the CD14^+^/CD16^+^-cells (44.9 to 19.0%) in the presence of statin was observed. In line with the hypothesis that GGPP may be involved in the differentiation of the macrophages, GGPP blocked the reversal of the CD14^+^/CD16^+^-expression to a large degree (Mac + GGPP; 44.9 vs. 28.5, compared to 44.9 vs. 19.0% in Mac). As expected, the addition of GGTI partially mimicked the statin effect, as shown by the low level (22.1%) of CD14^+^/CD16^+^-cells (No Stat; Mac + GGTI), and statin further reduced the CD14^+^/CD16^+^-cells in the GGTI-treated cells (13.5%; Stat; Mac + GGTI). In the cultures containing both GGPP and GGTI the data were similar. These results show that the expression of CD14^+^/CD16^−^ paralleled, and that the expression of CD14^+^/CD16^+^ inversely paralleled the retainment effect.

We also analyzed the expression of CD86, CCR2/CD192, CX_3_CR1, and CD163. Figure 6b shows that monocytes and macrophages expressed different levels of CD86; however, no apparent statin effect was noticeable. The expression of CCR2 in the monocytes also showed no statin-dependent differences. The macrophages expressed CCR2 less potent than the monocytes and statin further reduced the CCR2-level. Thus, both markers did not correlate with the retainment effect observed in cytokine production. On the other hand, the CX_3_CR1- and CD163-levels showed a clear correlation to the retainment data. Briefly, the monocyte curves with and without statin are more or less superimposing (blue and orange lines) and the fluorescence intensity of the macrophages prepared without statin was shifted to the right (black lines), indicating enhanced expression. In line with the hypothesis that the statin pretreatment may keep the “macrophages” in a more “monocytic” state, which is paralleled by the retainment of the inflammatory functions of these cells, the fluorescence levels of the statin-pretreated “macrophages” (red lines) were similar to those of the monocytes (blue and orange lines). Thus, in line with the enhanced level of IL-10 (compare Supplementary Fig. 4A) of macrophages pretreated without statin, the CX_3_CR1- and CD163-data suggest an anti-inflammatory phenotype of these macrophages, which does not develop in the presence of statin. The CD163 was expressed primarily in the CD14^+^-cells (Supplementary Fig. 4B). merTK, proposed to be preferentially found on macrophages, was enhanced in macrophages pretreated without statin (Supplementary Fig. 4C; black line); however, as described above for CX_3_CR1 and CD163, merTK-expression in statin-pretreated macrophages (red line) was comparable to that of monocytes.

Taken together, the data show that statins influence function(s) of monocytes and macrophages differentially. Geranylgeranylation of Rac1, but not regulation of NF-kB activity, is involved in the regulation of the retainment effect of statin in the macrophages. No such effect was observed in monocytes. These geranylgeranylation-regulated processes appear to be related to the differentiation status of the cells. These findings are of relevance for the comprehension of drug effects in multiple diseases, including auto-inflammatory diseases, sepsis, cancer, or atherosclerosis, which require contribution of monocytic cells, such as monocytes or macrophages.

## Discussion

**Fig. 7:**
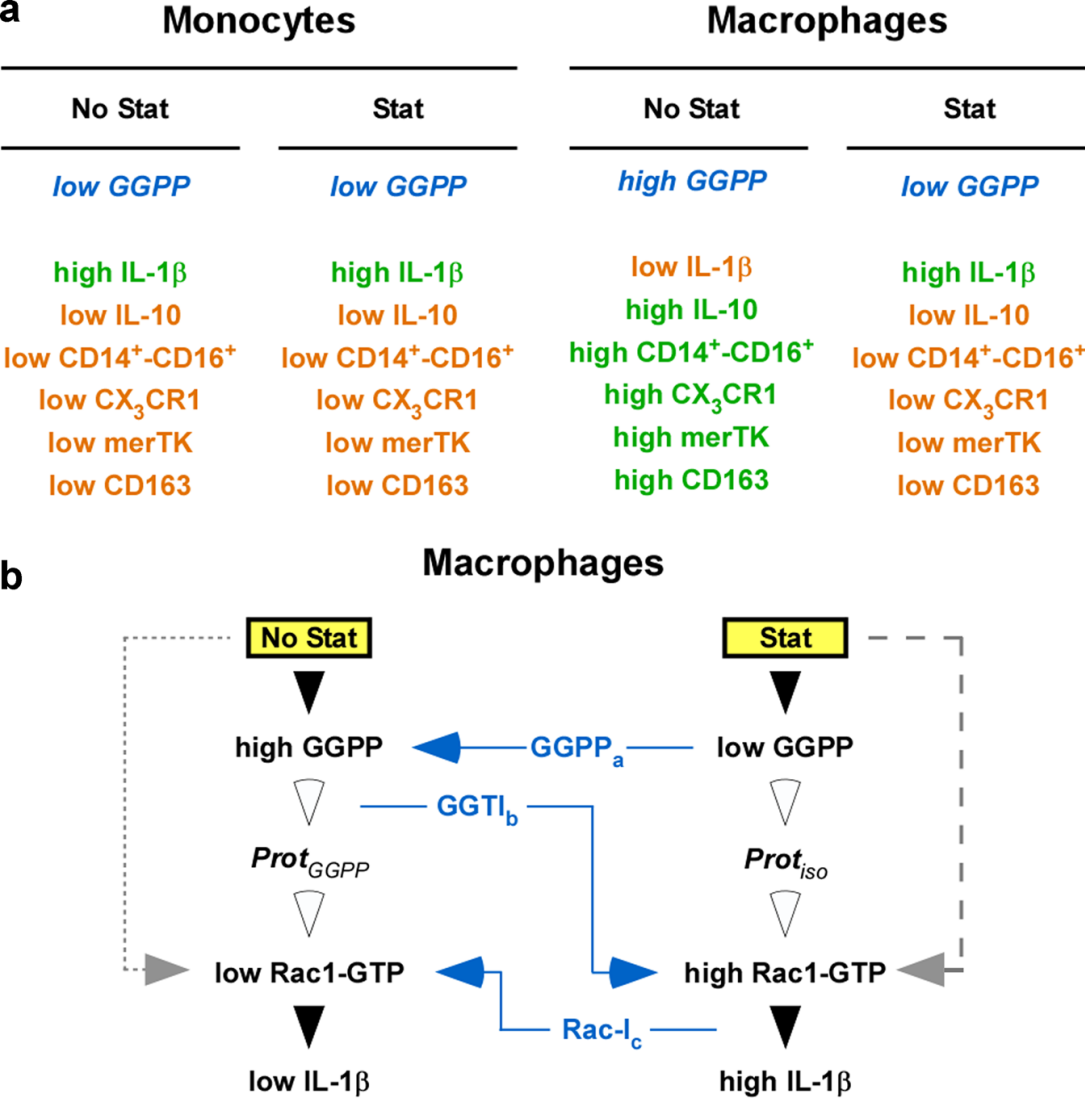
Hypothesis, summary, conclusion, and outlook. **a** Hypothesis and Summary—Monocytes and macrophages respond differentially to statin. Hypothesis: From our and other authors previous data we hypothesized that in Mo and Mac GGPP (blue/italic letters) might be present at different levels. Summary: This hypothesis appears to be correct, since in the present manuscript we show that statin does not significantly change the level of inflammatory cytokines in the monocytes. This was paralleled by a lack of changes in surface marker expression in the monocytes. However, we observed profound statin effects in overnight-differentiated macrophages. Green letters, high levels; orange letters, low levels. **b** Conclusion and Outlook—The statin effects on macrophages are geranylgeranylation-dependent. Conclusion: In the absence of statin (No Stat), high GGPP is present in the overnight-differentiated macrophages, which results in low Rac1-activation and low IL-1-levels. On the other hand, in the presence of statin (Stat) during the overnight differentiation, low GGPP is present, which results in enhanced Rac1- and subsequent high IL-1-levels^41^. This was proven by external GGPP and GGTI: a) addition of GGPP (GGPP_a_; blue letters; blue arrow) to the Mac treated with statin reversed statin’s effect. b) On the other hand, addition of GGTI (GGTI_b_; blue letters; blue arrow) to Mac prepared without statin blocked the geranylgeranylation, resulting in high Rac1 and subsequent high IL-1. c) Addition of a Rac1-inhibitor (Rac-I_c_; blue letters; blue arrow) to Mac prepared in the presence of statin may result in low Rac1-activity and, subsequently, in low IL-1-production. The open arrowheads indicate that the regulation pathway resulting in Rac1-activation is not yet defined. However, Rac1 may be regulated by some GEF, such as TIAM1, which may be determined in the future. Outlook: The retainment of function(s) in statin-treated Mac may be of importance in various diseases related to inflammatory processes, such as auto-inflammatory diseases, sepsis, cancer or atherosclerosis.

Previous publications described both pro- and anti-inflammatory actions of statins in monocytic cells^20–25,44–46^. We hypothesized that these varying results depended on differential isoprenylation effects in dissimilar monocytic phenotypes used, and that differential regulation of geranylgeranylation pathways, such as Rac1, in macrophages or monocytes may be involved. Thus, we compared the influence of statins on inflammatory cytokine production and differentiation of monocytes and macrophages. We show that cytokine production of LPS-stimulated, freshly isolated monocytes is not influenced by statin(s); however, the statins retained the cytokine production of overnight-differentiated macrophages. This retainment of cytokine production was paralleled by a retainment of monocyte markers in the statin-treated macrophages (summarized in Fig. 7a). These data are novel and they indicate that the statin alters the differentiation of the macrophages, rather than causing a costimulation, as believed previously. The retainment of the macrophages was Rac1-geranylgeranylation-dependent, as outlined in Fig. 7b.

We and others have reported anti-inflammatory effects of statins^20,44,45^. On the other hand, pro-inflammatory effects of statins have been suggested^21,46^. Since we hypothesized that the use of different cell phenotypes has caused the different results, we compared freshly isolated monocytes with overnight-differentiated macrophages. The latter frequently used in the mentioned literature. Summarizing the data of multiple experiments, we show that in freshly isolated monocytes the statistical analysis showed no significant effect of the statins. In sharp contrast, the effect of statin(s) on macrophages was enormous. Macrophages differentiated overnight in the absence of statin lost the capacity to produce cytokines; however, if differentiated overnight in the presence of statin, they produced “normal” levels of cytokines. This finding was evident at the protein, the mRNA, and the functional level. It was not limited to fluvastatin and the effect was time-dependent. Although previously suggested to be a costimulation effect, we show here in washing experiments that the presence of the statin is required during the differentiation phase, that is, in the first 24 h. However, if a costimulatory mechanism would be involved, the presence of statin during the stimulation phase should be sufficient to result in high IL-1 production, which, however, was not the case. Thus, we propose that the observed effect is rather a retainment effect. This indicates that the macrophages, differentiated in the presence of the statin, still keep a function or capacity to stay responsive to inflammatory activation, such as endotoxin stimulation.

The above-mentioned data suggest that differentiation of the cells is involved in the retainment of cytokine production. However, neither our previous study^20^ nor the other studies cited above^21–25^ had characterized the phenotype of the monocytic cells involved. Thus, we analyzed the expression of some surface markers. Basically, the expression of CD14/CD16, merTK, CX3CR1, and CD163 was shifted in the macrophages differentiated without statin, but was not shifted in the macrophages differentiated in the presence of statin. Complementing the cytokine data, this indicates that the statin may exert its influence on the cytokine production through the regulation of differentiation pathways in the cells. Enhanced levels of the anti-inflammatory cytokine IL-10 in the macrophages cultured without statin pointed in the same direction. Thus, the macrophages prepared without statin may represent a form of anti-inflammatory M2-cells. It has been suggested that merTK-positive cells are preferentially macrophages, but not monocytes, since circulating monocytes largely lack merTK, and merTK may be restricted to M2-macrophages^33^. In line with this suggestion and in addition to the CD14/16-, CX_3_CR1-, and CD163-data, the merTK/CD64-expression data presented here provide some evidence for the presence of macrophage characteristics in the macrophages prepared without statin, in contrast to “macrophages” prepared in the presence of statin. Our suggestion regarding the M1/M2-polarization is in line with other studies^45,47,48^.

Isoprenylation is an important regulator of various biological functions^49–52^. It has been reported previously that statin or GGTI can activate Rac1^53^ and that the enhanced Rac1-activity correlated with increased IL-1-levels^28^. Basically, our data are in line with these suggestions, since they show that GGPP is involved in the retainment of monocytic function(s) in the macrophages, and that the retainment is dependent on Rac1-geranylgeranylation. Our findings are also in line with data from Mandey et al.^25^, who showed that addition of GGPP to PBMC from MKD-patients reduced the IL-1 production. Possibly these cells reflected differentiated macrophages. The importance of geranylgeranylation of Rac1 was emphasized by the finding that the NF-kB-activity in stimulated monocytes or macrophages with and without statin, respectively, was not distinguishable. This finding was obtained in Western blot and p65-activation-assay. The data suggest that NF-kB is required for the response to LPS activation; however, other pathways, such as Rac1-regulation (or others), are important modulators of the inflammatory response. In the present cell culture model, the retainment effect caused by statin is modulated to a large degree by Rac1. Results of Li et al. are in line with our findings, since they suggest that in rat neutrophils and monocytes, the statin effect on matrix metallopeptidase-9 expression is independent of NF-kB or MAPK, but regulated by the GTPase RhoA^54^. Figuratively spoken: the car needs fuel (i.e., NF-kB) in order to be able to drive (i.e., to produce cytokines); however, the speed and the direction (pro- or anti-inflammatory) are determined by the gears, that is, by additional pathways, such as Rac1 and/or others. The regulation of the Rac1-activation in the macrophages may depend on GEF. Thus, the GEF Tiam1 may reflect a possible regulator of Rac1 in the present model, since the Rac1-inhibitor NSC23766, used in the present study, is supposed to interfere with the interaction of Rac1 and the GEF Tiam1^55,56^. In monocytes, on the other hand, the statin or addition of GGPP did not alter cytokine production. However, it remains open why the monocytes did not respond to statin. Possibly, they lack pathways present in macrophages, which interfere with the mevalonate pathway.

The present paper shows that statins sway monocytes and macrophages differentially, since statins have no significant effect on cytokine (IL-1, IL-6, TNF) production of monocytes, but keep macrophages from becoming hyporesponsive (retainment effect). The unchanged response of monocytes to statin is in line with a lack of change in ex vivo cytokine production before and after statin treatment of healthy volunteers^20,57^. The lack of response to statin in the present in vitro experiments may have been caused by an LPS-mediated interference with the mevalonate pathway, since there is information that inflammatory stress, such as by LPS or cytokines, may regulate HMG-CoA reductase activity or other parts of the mevalonate pathway^58,59^. However, this question was not addressed in the present paper.

Our results support the proposal of Wynn et al.^6^ that the mechanisms, which regulate the shift of pro- vs. anti-inflammatory, and even back again, are of great interest, because of their potential effect(s) during disease progression. The present data indicate that macrophages prepared in the presence of statin keep (retain) monocytic function(s) and stay in an immune/inflammatory-responsive state. This retainment of “immunocompetence” or “innatocompetence” may be responsible for some of the beneficial effects of statins observed in statin-treated patients.

## Materials and methods

### Materials

The used materials are described in the respective text. The antibodies used in this study are listed in Supplementary Table 1.

### Monocyte isolation and cell culture

On day 1, mononuclear cells (MNC) were isolated from heparinized buffy coats, provided by the “Einrichtung für Transfusionsmedizin” (Universitätsklinikum Halle (Saale)). The voluntary donors gave written informed consent. The use of the cells was approved by the local ethical committee. The buffy coat was mixed with the same volume of phosphate-buffered saline (PBS; Biochrom GmbH, Berlin, Germany) and applied to gradient centrifugation using Biocoll (30 min, 400 × *g*; no acceleration, no deceleration; Biochrom). The obtained cells were washed twice (200 × *g*, 10 min) in VLE-RPMI-1640, containing 1% penicillin/streptomycin and 1% l-glutamine (all Biochrom).

Monocytes were prepared from the MNC using CD14-MicroBeads (Miltenyi Biotec GmbH, Bergisch Gladbach, Germany). The monocytes (Fig. 1a, left part) were incubated in the absence (M) or presence (S) of fluvastatin (Calbiochem, Merck KGaA, Darmstadt, Germany) and without (N) or with (L) LPS (Glycobiotech, Kükels, Germany). On day 2, the monocyte supernatants and/or cells (SN-IC_Mo_) were harvested for the respective analyses.

In order to obtain monocyte-derived macrophages, the freshly isolated monocytes were incubated for 24 h in the absence or presence of statin (Fig. 1a, right part). On day 2, LPS or medium without LPS was added and the macrophage cultures were incubated for further 24 h. On day 3, the macrophage supernatants and/or cells were harvested for the respective analyses (SN-IC_Mac_).

### Cytokine ELISAs

Enzyme-linked immunosorbent assays (OptEIA ELISA for IL-1β, IL-6, IL-10, or TNFα) were performed according to the manufacturer’s instructions (BD Biosciences, Heidelberg, Germany).

### Biological IL-1 determination with the fibroblast assay

Biological interleukin-1 activity was determined as described previously^40^. Briefly, human skin fibroblasts (5000 cells/well; 100 µl/well) were cultured overnight in 96-well plates (Nunc, Thermo Fisher Scientific). The medium was aspirated and replaced by fresh medium (Dulbecco’s modified Eagle’s medium with 4.5 g/l glucose (Biochrom), containing 1% l-glutamine, 1% antibiotics, and 10% fetal calf serum (Thermo Fisher Scientific, Schwerte, Germany). Serial 1:4 dilutions of standard (recombinant IL-1α, 10 ng/ml; PeproTech, Hamburg, Germany) or samples were performed and cultured for 96 h. The cell layers were washed, fixed with 3% formaldehyde (Sigma-Aldrich, Merck KGaA, Darmstadt, Germany), and stained with crystal violet (Serva Electrophoresis GmbH, Heidelberg, Germany). The absorption was read at 540 nm in an ELISA reader (Spectrafluor, Tecan, Crailsheim, Germany), the data plotted and the ED_50_ (median effective dose) determined. The biological activity (U/ml) was calculated with respect to the recombinant standard (10 U/ml ≙ 10 ng/ml recombinant IL-1α).

### Rac1 pull-down assay

For Rac1-activation analysis, the cells were washed with PBS and subsequently lysed in cell lysis buffer, provided with the “Rac1 Activation Assay Biochem Kit” (Cytoskeleton, Denver, USA). The protein concentration was determined using the “BCA Protein Assay Kit” (Pierce, Thermo Fisher Scientific) and 350 µg of the cell lysates was incubated at 4 °C with 10 µl of the “PAK-PBD beads” on a rotator for 1 h. The “PAK-PBD beads” were pelleted (1 min, 4 °C, 5000 × *g*), the supernatants carefully removed, and analyzed for glyceraldehyde 3-phosphate dehydrogenase (GAPDH) and total Rac1 in Western blot. The beads, carrying the specifically bound Rac1-GTP, were resuspended with 500 µl wash buffer. The beads were again pelleted (3 min, 4 °C, 5000 × *g*), the pellets resuspended in water, sample buffer added, and the samples analyzed in Western blot. The measurement of RhoA was performed accordingly, also as suggested by the manufacturer (Cytoskeleton).

### TransAM assay

In order to measure NF-kB- and relB-activation, monocytes were incubated for 20 min with and without LPS, in the absence or presence of statin. Macrophages were cultured with or without statin or statin and GGPP for 24 h and then incubated for 20 min with LPS. Subsequently, the nuclear fraction and the cytosol were isolated using the “Nuclear Extraction Kit” (Active Motif), according to the manufacturer’s instructions. The samples were analyzed in standard sodium dodecyl sulfate-polyacrylamide gel electrophoresis (SDS-PAGE) and silver staining, and equal amounts were applied to the “TransAM® NF-kB Family Transcription Factor Assay Kit” (Active Motif). The NF-kB p65 and RelB of the samples were then measured according to the manufacturer’s instructions.

### Western blot

Samples were incubated for 3 min at 95 °C in sample buffer (63 mM Tris base, pH 6.8; 10% glycerol, 2% SDS, 0.01% (w/v) bromphenolblue, 0.5% β-mercaptoethanol). The proteins were separated by standard SDS-PAGE (12%) and transferred onto a nitrocellulose blotting membrane (0.45 µm; Amersham™ Protran; GE Healthcare, Freiburg, Germany) by electroblotting (1 mA/cm^2^, 1.5 h). The membranes were blocked using 5% bovine serum albumin (BSA) in TBS-T (BSA, fraction V; Carl Roth GmbH, Karlsruhe, Germany; 1 h, 4 °C; TBS-T consists of 20 mM Tris-HCl, 150 mM NaCl (pH 7.5), and 0.1% Tween-20). The blots were incubated overnight with the first antibody (4 °C; BSA/TBS-T). After three washing steps (TBS-T), the blots were incubated with the respective secondary antibody for 1 h at room temperature and again washed three times. Data analysis was performed using the “Super Signal™ West Dura Extended Duration Substrate” (Thermo Fisher Scientific) in the “ImageQuant LAS 4000” (GE Healthcare). Density analysis was performed using the TotalLab Quant software (TotalLab Limited, Newcastle, England).

### RNA isolation, reverse transcription, qPCR, and microRNA array

Total RNA was extracted with the “RNeasy Plus Mini Kit” (Qiagen, Hilden, Germany) according to the manufacturer’s instructions and used for deep sequencing (compare Supplementary Table 2) or qPCR. Reverse transcription and qPCR were performed using one of the following protocols.

#### Omniscript protocol

For use with the “Omniscript Reverse Transcription Kit” (Qiagen), RNA was adjusted to 100 ng per reaction in diethyl pyrocarbonate (DEPC)-treated water (total volume of 12 µl; Fermentas, Thermo Fisher Scientific). To the RNA, the kit components RT-buffer (2 µl), dNTP-mix (2 µl; 0.5 mM), and “Omniscript Reverse Transcriptase” (1 µl; 4 U), as well as separately obtained “Rnasin Plus Rnase Inhibitor” (1 µl; 10 U; Promega GmbH, Mannheim, Germany) and oligo(dT)18 primer (2 µl; 1 µM; Fermentas) were added (total volume of 20 µl). The reaction was performed at 37 °C for 60 min and DEPC-treated water (80 µl) was added. Real-time PCR was conducted in a volume of 25 µl consisting of 2.5 µl cDNA, 12.5 µl “GoTaq qPCR Master Mix” (Promega; including the proprietary double-strand DNA stain), 0.5 µl sense primer, 0.5 µl antisense primer (0.25 µM each), and 9 µl DEPC-treated water. The reaction was performed using the Bio-Rad “iCycler iQ PCR Detection System” (Bio-Rad Laboratories, Munich, Germany). The IL-6 primers were 5′-TCGGTACATCCTCGACGGCA-3′ (sense) and 5′-TCACCAGGCAAGTCTCCTCA-3′ (antisense); the IL-1β primers were 5′-ACAAGGCACAACAGGCTGCTC-3′ (sense) and 5′-GGTCCTGGAAGGAGCACTTCAT-3′ (antisense); the GAPDH primers were 5′-AGGGCTGCTTTTAACTCTGGT-3′ (sense) and 5′-CCCCACTTGATTTTGGAGGGA-3′ (antisense). The primers were obtained from MWG Biotech AG (Ebersberg, Germany). For quantification, the IL-1- or IL-6-mRNA expression was normalized to GAPDH.

#### iScript protocol

For use with the “iScript™ cDNA Synthesis Kit” (Bio-Rad Laboratories), RNA (75–200 ng) was diluted in DEPC-treated water (15 µl; kit contents). To this, 4 µl RT-buffer (5-fold) and 1 µl reverse transcriptase was added (both in the kit). The reaction was performed for 40 min according to the manufacturer’s instructions and DEPC-treated water (180 µl) was added. For PCR, the “TaqMan® Gene Expression Master Mix” (Applied Biosystems, Thermo Fisher Scientific) was used: 12.5 µl of the master mix (2-fold), 1.25 µl of the respective “TaqMan® Gene Expression Assay” (20-fold), and 6.25 µl DEPC-treated water were added to the wells of a 96-well plate. Samples or controls (5 µl each) were added to triplicate wells and the plates run as suggested by the manufacturer. The following “TaqMan® Gene Expression Assays” were used: IL-1β, hs00174097; IL-6, hs00985639; GAPDH, hs02758991. For quantification, the IL-1- or IL-6-mRNA expression was normalized to GAPDH.

#### microRNA protocol

For analysis of microRNA expression, total RNA was isolated as described above. The microRNAs were quantified in PCR using the “TaqMan® MicroRNA Reverse Transcription Kit” (Applied Biosystems) and the “TaqMan® MicroRNA Assays” (Applied Biosystems). For reverse transcription, the RNA was adjusted to 10 ng in 3 µl DEPC-treated water and added to 12 µl of the reaction mix, which was prepared from compounds of the kit. Per 12 µl the reaction mix consisted of dNTPs (100 mM, each; 0.3 µl), “MultiScribe™ Reverse Transcriptase” (3.0 µl), “Reverse Transcription Buffer” (1.5 µl), RNAse inhibitor (0.192 µl), primer pool (6.0 µl), and DEPC-treated water (1.008 µl). The primer pool used in the reaction mix was prepared by adding 10 µl of the respective RT-primers delivered with the “TaqMan® MicroRNA Assays” to 1000 µl DEPC-treated water. Fifteen microliters (3 µl RNA, 12 µl reaction mix) of the sample was incubated for 30 min at 16 °C, for 30 min at 42 °C, for 5 min at 85 °C, and finally at 4 °C. To each of the cDNA-samples, 35 µl of DEPC-treated water was added and the samples were stored at −20 °C. For qPCR, the cDNA-sample (1.33 µl), the respective PCR primer delivered with the “TaqMan^®^ MicroRNA Assays” (1 µl), the “TaqMan Universal Master Mix II” (10 µl; Applied Biosystems), and 7.67 µl DEPC-treated water was incubated (total of 20 µl) in 96-well plates (1 cycle: 10 min, 95 °C; 40 cycles: 15 s 95 °C and 60 s 60 °C; 1 cycle: 60 °C) in the “7500 Real-Time PCR System” (Applied Biosystems). The microRNA expression was normalized to the U6 housekeeping gene (Applied Biosystems).

### Transfection experiments

“Lipofectamine® 2000” (ambion™, Invitrogen, Thermo Fisher Scientific) and siRNAs (ambion™) were diluted separately in “OptiMEM®” (Gibco™, Life Technologies, Thermo Fisher Scientific). After 5 min, equal volumes were mixed and incubated for 20 min at room temperature. Subsequently, 250 µl of the mix was added to 2250 µl cell suspension in 6-well plates (Nunc) for 24 h. LPS (100 ng/ml) was then added for further 24 h. The supernatants were analyzed in ELISA and the cell lysates in Western blot.

The contribution of microRNAs was investigated using “miRCURY LNA™” anti-miR’s. “Lipofectamine® RNAiMAX” (9 µl; ambion™) was added to 150 µl of “OptiMEM®” and 3 µl of the anti-miR (10 µM) was added to another volume of 150 µl “Optimem®”. Subsequently, both preparations were mixed and incubated for 5 min, applied to the cultures (250 µl mix plus 2750 µl culture; 6-well plate), incubated for 24 h, LPS was added, and the cells were cultured for additional 24 h.

### Fluorescence-activated cell sorting

FACS-analysis was performed as explained in the respective legends. At least 5000 cells per sample were analyzed. The basic gating strategy is shown in an exemplary way in Supplementary Fig. 5A–D. The cells were defined positive for a surface marker if the expression level was higher than that of the isotype control. As shown in Supplementary Fig. 5E, the statin did not change the viability of the cells.

### Statistical methods

ELISA and fibroblast assay measurements were performed in triplicates. These multiple values were used to calculate the mean and the standard deviation, as outlined in the respective figure legends. Significances were calculated using SPSS (Levene’s test; one-way analysis of variance (ANOVA); Welch’s ANOVA; Games–Howell or least significant difference post hoc analysis), as described in the respective figure legends.

## Supplementary information

supplemental tables

supplemental legends

supplemental Figure 1A-C

supplemental Figure 1D-G

supplemental Figure 2

supplemental Figure 3

supplemental Figure 4

supplemental Figur 5
