# Supplementary material for: The differential statin effect on cytokine production of monocytes or macrophages is mediated by differential geranylgeranylation-dependent Rac1 activation
Source: Cell Death Dis. 2019 Nov 21;10(12):880. doi: 10.1038/s41419-019-2109-9 (PMC6872739; doi:10.1038/s41419-019-2109-9)
Supplement: Supplementary file 1 — supplemental tables [file 41419_2019_2109_MOESM1_ESM.docx]

| **Supplement Table 1. Antibodies used in the experiments.** | | | | | | | |
| --- | --- | --- | --- | --- | --- | --- | --- |
| **Antigen** | **Clone** | **Class** | **Source** | **Company** | **Order no.** | **Stain** | **^a^Assay** |
| IL-1α | #4414 | IgG2A | mouse | R+D | MAB200 |  | Inhib |
| IL-1β |  | IgG | goat | R+D | AF-201-NA |  | Inhib |
| ^b^IL-1β | FIB1 | IgG1 | mouse |  |  |  | WB |
| GAPDH |  | IgG | goat | R+D | AF5718 |  | WB |
| Caspase‑1 |  | IgG | rabbit | Santa Cruz | sc-515 |  | WB |
| Rac1 | C7H2 |  | mouse | Cytoskeleton | ARC03 (BK035) |  | WB/PD |
| anti-rabbit IgG |  |  | donkey | dianova | 711-035-152 | HRP | WB |
| anti-goat IgG |  |  | donkey | Santa Cruz | sc-2020 | HRP | WB |
| anti-mouse IgG |  |  | goat | dianova | 115-036-071 | HRP | WB |
| p-38 |  |  | rabbit | Cell Signaling | 9212 |  | WB |
| p-p38 |  |  | rabbit | Cell Signaling | 9211 |  | WB |
| NF‑kB |  | IgG | rabbit | Cell Signaling | 8242 |  | WB |
| p-NF‑kB |  | IgG | rabbit | Cell Signaling | 3033 |  | WB |
| I‑kB | L35A5 | IgG1 | mouse | Cell Signaling | 4814 |  | WB |
| p‑I‑kB | 5A5 | IgG1 | mouse | Cell Signaling | 9246 |  | WB |
| CD14 | TÜK4 | IgG2A | mouse | Miltenyi | 130-080-701 | FITC | FACS |
| ^c^iso | S43.10 | IgG2A | mouse | Miltenyi | 130-091-837 | FITC | FACS |
| CD16 | 3G8 | IgG1 | mouse | biolegend | 302016 | PE/Cy7 | FACS |
| iso | MOPC-21 | IgG1 | mouse | biolegend | 400126 | PE/Cy7 | FACS |
| CCR2/CD192 | K036C2 | IgG2A | Mouse | biolegend | 357206 | PE | FACS |
| iso | MOPC-173 | IgG2A | Mouse | biolegend | 400214 | PE | FACS |
| CD86 | 2331 (FUN-1) | IgG1 | mouse | BD | 555658 | PE | FACS |
| iso | MOPC-21 | IgG1 | mouse | BD | 556650 | PE | FACS |
| CX_3_CR1 | 2A9-1 | IgG2B | rat | biolegend | 341614 | PerCP/Cy5.5 | FACS |
| iso | RTK4530 | IgG2B | rat | biolegend | 400632 | PerCP/Cy5.5 | FACS |
| CD163 | GHI/61 | IgG1 | mouse | biolegend | 333610 | APC | FACS |
| iso | MOPC-21 | IgG1 | mouse | biolegend | 400120 | APC | FACS |
| a - Inhib, inhibition of the respective biological IL‑1-activity by the antibody was analyzed in the fibroblast assay^1^; WB, Western blot; PD, pull-down-assay; FACS, fluorescence-activated cell-sorting.  b - Compare references Blum et al. and Herzbeck et al.^2, 3^.  c - iso, isotype control for the afore-mentioned respective antibody. | | | | | | | |

| **Supplement Table 2. Down- or up-regulated microRNAs in LPS-stimulated macrophages derived in the presence of statin, as compared to LPS-stimulated macrophages derived in the absence of statin.** | | | | | | |
| --- | --- | --- | --- | --- | --- | --- |
|  | ^a^Mac  (counts) | |  |  |  |  |
| microRNA | Statin  (A) | No statin  (B) | ^b^Mean_AB_ | Lg2_MeanAB_ | ^c^A/B | ^d^Lg2_A/B_ |
| hsa-miR-146b-5p | 12929 | 51056 | 31992 | 14.97 | 0.25 | -1.981 |
| hsa-miR-155-5p | 7899 | 25220 | 16560 | 14.02 | 0.31 | -1.675 |
| hsa-miR-21-3p | 1878 | 4288 | 3083 | 11.59 | 0.44 | -1.191 |
| hsa-miR-23b-3p | 68 | 144 | 106 | 6.73 | 0.47 | -1.078 |
| hsa-miR-146a-5p | 12246 | 25076 | 18661 | 14.19 | 0.49 | -1.034 |
| hsa-miR-3065-5p | 91 | 148 | 120 | 6.90 | 0.61 | -0.713 |
| hsa-miR-338-3p | 91 | 148 | 120 | 6.90 | 0.61 | -0.713 |
| hsa-mir-3065 | 101 | 164 | 132 | 7.05 | 0.62 | -0.695 |
| hsa-mir-338 | 101 | 164 | 132 | 7.05 | 0.62 | -0.695 |
| hsa-miR-99b-5p | 2095 | 3390 | 2742 | 11.42 | 0.62 | -0.695 |
| hsa-miR-7-5p | 186 | 295 | 240 | 7.91 | 0.63 | -0.664 |
| hsa-miR-125a-5p | 686 | 1062 | 874 | 9.77 | 0.65 | -0.631 |
| hsa-mir-7-1 | 80 | 123 | 101 | 6.67 | 0.65 | -0.619 |
| hsa-miR-9-5p | 93 | 137 | 115 | 6.85 | 0.67 | -0.567 |
| hsa-let-7i-5p | 15338 | 22029 | 18683 | 14.19 | 0.70 | -0.522 |
| hsa-mir-21 | 33078 | 43879 | 38479 | 15.23 | 0.75 | -0.408 |
| hsa-miR-345-5p | 841 | 1100 | 970 | 9.92 | 0.76 | -0.388 |
| hsa-mir-29a | 379 | 495 | 437 | 8.77 | 0.76 | -0.387 |
| hsa-miR-186-5p | 3572 | 4669 | 4121 | 12.01 | 0.77 | -0.386 |
| hsa-miR-29a-3p | 379 | 493 | 436 | 8.77 | 0.77 | -0.382 |
| hsa-mir-454 | 162 | 209 | 185 | 7.53 | 0.77 | -0.372 |
| hsa-let-7c-5p | 120 | 154 | 137 | 7.10 | 0.78 | -0.366 |
| hsa-miR-454-3p | 152 | 193 | 172 | 7.43 | 0.78 | -0.349 |
| hsa-miR-21-5p | 31168 | 39501 | 35334 | 15.11 | 0.79 | -0.342 |
| hsa-let-7f-2 | 22707 | 28461 | 25584 | 14.64 | 0.80 | -0.326 |
| hsa-let-7f-5p | 43582 | 54439 | 49011 | 15.58 | 0.80 | -0.321 |
| hsa-mir-660 | 141 | 176 | 158 | 7.31 | 0.80 | -0.316 |
| hsa-let-7f-1 | 20883 | 25985 | 23434 | 14.52 | 0.80 | -0.315 |
| hsa-miR-660-5p | 141 | 175 | 158 | 7.30 | 0.80 | -0.315 |
| hsa-mir-185 | 242 | 296 | 269 | 8.07 | 0.82 | -0.293 |
| hsa-mir-15a | 266 | 325 | 296 | 8.21 | 0.82 | -0.286 |
| hsa-miR-15a-5p | 266 | 325 | 296 | 8.21 | 0.82 | -0.286 |
| hsa-miR-185-5p | 224 | 271 | 248 | 7.95 | 0.82 | -0.279 |
| hsa-mir-16-1 | 11552 | 13920 | 12736 | 13.64 | 0.83 | -0.269 |
| hsa-mir-101-1 | 108 | 129 | 119 | 6.89 | 0.83 | -0.261 |
| hsa-miR-16-5p | 23189 | 27742 | 25465 | 14.64 | 0.84 | -0.259 |
| hsa-miR-361-3p | 158 | 188 | 173 | 7.44 | 0.84 | -0.254 |
| hsa-mir-16-2 | 11720 | 13907 | 12814 | 13.65 | 0.84 | -0.247 |
| hsa-miR-130b-3p | 198 | 230 | 214 | 7.74 | 0.86 | -0.212 |
| hsa-mir-769 | 138 | 160 | 149 | 7.22 | 0.87 | -0.205 |
| hsa-mir-151a | 256 | 295 | 276 | 8.11 | 0.87 | -0.204 |
| hsa-let-7e-5p | 1569 | 1804 | 1686 | 10.72 | 0.87 | -0.202 |
| hsa-miR-769-5p | 137 | 158 | 148 | 7.21 | 0.87 | -0.202 |
| hsa-let-7e | 1579 | 1808 | 1693 | 10.73 | 0.87 | -0.196 |
| hsa-miR-151a-3p | 198 | 225 | 212 | 7.72 | 0.88 | -0.185 |
| hsa-miR-30b-5p | 366 | 415 | 391 | 8.61 | 0.88 | -0.182 |
| hsa-let-7a-1 | 26873 | 30199 | 28536 | 14.80 | 0.89 | -0.168 |
| hsa-miR-98-5p | 4942 | 5554 | 5248 | 12.36 | 0.89 | -0.168 |
| hsa-mir-98 | 4945 | 5555 | 5250 | 12.36 | 0.89 | -0.168 |
| hsa-miR-181c-5p | 518 | 581 | 549 | 9.10 | 0.89 | -0.167 |
| hsa-miR-101-3p | 237 | 266 | 251 | 7.97 | 0.89 | -0.166 |
| hsa-miR-181d-5p | 105 | 118 | 112 | 6.80 | 0.89 | -0.166 |
| hsa-miR-28-3p | 1072 | 1201 | 1136 | 10.15 | 0.89 | -0.164 |
| hsa-let-7a-5p | 80759 | 90219 | 85489 | 16.38 | 0.90 | -0.160 |
| hsa-mir-181d | 106 | 118 | 112 | 6.81 | 0.90 | -0.159 |
| hsa-mir-181c | 533 | 595 | 564 | 9.14 | 0.90 | -0.159 |
| hsa-let-7a-2 | 26748 | 29786 | 28267 | 14.79 | 0.90 | -0.155 |
| hsa-let-7a-3 | 27167 | 30245 | 28706 | 14.81 | 0.90 | -0.155 |
| hsa-miR-22-3p | 12185 | 13455 | 12820 | 13.65 | 0.91 | -0.143 |
| hsa-mir-22 | 12199 | 13465 | 12832 | 13.65 | 0.91 | -0.142 |
| hsa-mir-374b | 158 | 174 | 166 | 7.37 | 0.91 | -0.140 |
| hsa-mir-374c | 158 | 174 | 166 | 7.37 | 0.91 | -0.140 |
| hsa-mir-101-2 | 129 | 141 | 135 | 7.07 | 0.92 | -0.123 |
| hsa-mir-181b-2 | 2161 | 2349 | 2255 | 11.14 | 0.92 | -0.120 |
| hsa-miR-1307-5p | 643 | 699 | 671 | 9.39 | 0.92 | -0.120 |
| hsa-let-7d-5p | 3478 | 3778 | 3628 | 11.83 | 0.92 | -0.119 |
| hsa-mir-28 | 1403 | 1519 | 1461 | 10.51 | 0.92 | -0.115 |
| hsa-miR-374b-5p | 154 | 166 | 160 | 7.32 | 0.93 | -0.111 |
| hsa-miR-374c-3p | 154 | 166 | 160 | 7.32 | 0.93 | -0.111 |
| hsa-mir-484 | 323 | 347 | 335 | 8.39 | 0.93 | -0.106 |
| hsa-mir-130b | 256 | 275 | 266 | 8.05 | 0.93 | -0.103 |
| hsa-miR-181b-5p | 4295 | 4601 | 4448 | 12.12 | 0.93 | -0.099 |
| hsa-miR-30e-5p | 4000 | 4271 | 4135 | 12.01 | 0.94 | -0.095 |
| hsa-let-7d | 3681 | 3929 | 3805 | 11.89 | 0.94 | -0.094 |
| hsa-mir-652 | 112 | 119 | 115 | 6.85 | 0.94 | -0.092 |
| hsa-let-7g-5p | 19418 | 20648 | 20033 | 14.29 | 0.94 | -0.089 |
| hsa-mir-421 | 259 | 275 | 267 | 8.06 | 0.94 | -0.087 |
| hsa-mir-30e | 4924 | 5227 | 5075 | 12.31 | 0.94 | -0.086 |
| hsa-mir-181b-1 | 2135 | 2252 | 2193 | 11.10 | 0.95 | -0.077 |
| hsa-miR-142-3p | 170 | 179 | 174 | 7.45 | 0.95 | -0.072 |
| hsa-miR-10a-5p | 468 | 492 | 480 | 8.91 | 0.95 | -0.071 |
| hsa-miR-652-3p | 110 | 115 | 112 | 6.81 | 0.95 | -0.070 |
| hsa-mir-10a | 469 | 492 | 480 | 8.91 | 0.95 | -0.070 |
| hsa-mir-24-2 | 518 | 542 | 530 | 9.05 | 0.96 | -0.065 |
| hsa-miR-192-5p | 233 | 243 | 238 | 7.90 | 0.96 | -0.062 |
| hsa-miR-421 | 257 | 267 | 262 | 8.03 | 0.96 | -0.053 |
| hsa-miR-30e-3p | 923 | 955 | 939 | 9.87 | 0.97 | -0.049 |
| hsa-miR-148b-3p | 3061 | 3147 | 3104 | 11.60 | 0.97 | -0.040 |
| hsa-mir-361 | 432 | 444 | 438 | 8.78 | 0.97 | -0.038 |
| hsa-mir-181a-1 | 22108 | 22656 | 22382 | 14.45 | 0.98 | -0.035 |
| hsa-miR-142-5p | 13111 | 13414 | 13263 | 13.70 | 0.98 | -0.033 |
| hsa-miR-328-3p | 106 | 109 | 107 | 6.75 | 0.98 | -0.030 |
| hsa-miR-181a-5p | 44266 | 44897 | 44582 | 15.44 | 0.99 | -0.020 |
| hsa-miR-24-3p | 956 | 968 | 962 | 9.91 | 0.99 | -0.018 |
| hsa-mir-103a-1 | 2508 | 2535 | 2522 | 11.30 | 0.99 | -0.015 |
| hsa-mir-103b-1 | 2508 | 2535 | 2522 | 11.30 | 0.99 | -0.015 |
| hsa-mir-340 | 897 | 901 | 899 | 9.81 | 1.00 | -0.007 |
| hsa-mir-181a-2 | 22260 | 22346 | 22303 | 14.44 | 1.00 | -0.006 |
| hsa-miR-340-5p | 852 | 855 | 854 | 9.74 | 1.00 | -0.004 |
| hsa-mir-20a | 160 | 160 | 160 | 7.32 | 1.00 | -0.001 |
| hsa-miR-103a-3p | 4957 | 4954 | 4955 | 12.27 | 1.00 | 0.001 |
| hsa-miR-103b | 4957 | 4954 | 4955 | 12.27 | 1.00 | 0.001 |
| hsa-mir-26b | 7179 | 7174 | 7176 | 12.81 | 1.00 | 0.001 |
| hsa-miR-26b-5p | 7163 | 7143 | 7153 | 12.80 | 1.00 | 0.004 |
| hsa-miR-20a-5p | 160 | 158 | 159 | 7.31 | 1.01 | 0.013 |
| hsa-mir-1307 | 857 | 848 | 852 | 9.74 | 1.01 | 0.016 |
| hsa-mir-103b-2 | 2453 | 2424 | 2439 | 11.25 | 1.01 | 0.017 |
| hsa-miR-3074-5p | 485 | 479 | 482 | 8.91 | 1.01 | 0.019 |
| hsa-mir-103a-2 | 2468 | 2434 | 2451 | 11.26 | 1.01 | 0.020 |
| hsa-mir-24-1 | 486 | 479 | 482 | 8.91 | 1.01 | 0.021 |
| hsa-mir-3074 | 486 | 479 | 482 | 8.91 | 1.01 | 0.021 |
| hsa-let-7b-5p | 2084 | 2036 | 2060 | 11.01 | 1.02 | 0.034 |
| hsa-miR-221-3p | 3099 | 3008 | 3054 | 11.58 | 1.03 | 0.043 |
| hsa-miR-191-5p | 19837 | 19236 | 19537 | 14.25 | 1.03 | 0.044 |
| hsa-mir-15b | 354 | 342 | 348 | 8.44 | 1.03 | 0.047 |
| hsa-miR-28-5p | 331 | 319 | 325 | 8.34 | 1.04 | 0.054 |
| hsa-miR-425-5p | 1717 | 1644 | 1680 | 10.71 | 1.04 | 0.063 |
| hsa-mir-374a | 150 | 143 | 146 | 7.19 | 1.05 | 0.071 |
| hsa-mir-532 | 379 | 361 | 370 | 8.53 | 1.05 | 0.071 |
| hsa-mir-425 | 1779 | 1691 | 1735 | 10.76 | 1.05 | 0.073 |
| hsa-miR-15b-5p | 348 | 331 | 339 | 8.41 | 1.05 | 0.075 |
| hsa-miR-532-5p | 365 | 346 | 355 | 8.47 | 1.06 | 0.078 |
| hsa-miR-223-5p | 134 | 125 | 130 | 7.02 | 1.07 | 0.094 |
| hsa-mir-27b | 1124 | 1049 | 1087 | 10.09 | 1.07 | 0.100 |
| hsa-miR-342-3p | 354 | 331 | 342 | 8.42 | 1.07 | 0.101 |
| hsa-miR-27b-3p | 1124 | 1048 | 1086 | 10.09 | 1.07 | 0.101 |
| hsa-miR-361-5p | 274 | 255 | 265 | 8.05 | 1.07 | 0.103 |
| hsa-mir-26a-2 | 22375 | 20793 | 21584 | 14.40 | 1.08 | 0.106 |
| hsa-mir-342 | 401 | 371 | 386 | 8.59 | 1.08 | 0.112 |
| hsa-miR-26a-5p | 44927 | 41512 | 43220 | 15.40 | 1.08 | 0.114 |
| hsa-miR-107 | 428 | 394 | 411 | 8.68 | 1.09 | 0.118 |
| hsa-mir-26a-1 | 22552 | 20722 | 21637 | 14.40 | 1.09 | 0.122 |
| hsa-miR-374a-5p | 131 | 120 | 125 | 6.97 | 1.09 | 0.125 |
| hsa-miR-378a-3p | 920 | 835 | 878 | 9.78 | 1.10 | 0.140 |
| hsa-miR-148a-3p | 1722 | 1558 | 1640 | 10.68 | 1.11 | 0.145 |
| hsa-miR-30c-5p | 2055 | 1852 | 1954 | 10.93 | 1.11 | 0.150 |
| hsa-mir-30c-2 | 1005 | 905 | 955 | 9.90 | 1.11 | 0.150 |
| hsa-mir-30c-1 | 1065 | 958 | 1012 | 9.98 | 1.11 | 0.153 |
| hsa-miR-23a-3p | 1074 | 961 | 1018 | 9.99 | 1.12 | 0.160 |
| hsa-miR-500a-3p | 579 | 517 | 548 | 9.10 | 1.12 | 0.165 |
| hsa-miR-320a | 1592 | 1419 | 1506 | 10.56 | 1.12 | 0.166 |
| hsa-mir-23a | 1084 | 964 | 1024 | 10.00 | 1.12 | 0.169 |
| hsa-miR-140-3p | 2059 | 1818 | 1938 | 10.92 | 1.13 | 0.180 |
| hsa-mir-17 | 328 | 287 | 308 | 8.27 | 1.14 | 0.191 |
| hsa-miR-501-3p | 494 | 426 | 460 | 8.85 | 1.16 | 0.214 |
| hsa-miR-589-5p | 120 | 103 | 111 | 6.80 | 1.16 | 0.215 |
| hsa-miR-93-5p | 2429 | 2054 | 2241 | 11.13 | 1.18 | 0.242 |
| hsa-miR-143-3p | 262 | 220 | 241 | 7.91 | 1.19 | 0.251 |
| hsa-miR-19b-3p | 142 | 118 | 130 | 7.02 | 1.20 | 0.262 |
| hsa-miR-199a-3p | 191 | 159 | 175 | 7.45 | 1.20 | 0.263 |
| hsa-mir-106b | 348 | 289 | 318 | 8.31 | 1.20 | 0.266 |
| hsa-mir-199b | 162 | 132 | 147 | 7.20 | 1.23 | 0.295 |
| hsa-miR-363-3p | 227 | 185 | 206 | 7.69 | 1.23 | 0.296 |
| hsa-miR-3184-5p | 1670 | 1354 | 1512 | 10.56 | 1.23 | 0.302 |
| hsa-miR-423-3p | 1670 | 1354 | 1512 | 10.56 | 1.23 | 0.302 |
| hsa-miR-744-5p | 470 | 378 | 424 | 8.73 | 1.24 | 0.312 |
| hsa-miR-17-5p | 280 | 222 | 251 | 7.97 | 1.26 | 0.336 |
| hsa-miR-150-5p | 783 | 619 | 701 | 9.45 | 1.26 | 0.339 |
| hsa-mir-150 | 797 | 627 | 712 | 9.48 | 1.27 | 0.346 |
| hsa-mir-941-1 | 202 | 158 | 180 | 7.49 | 1.28 | 0.355 |
| hsa-mir-92a-1 | 24395 | 18865 | 21630 | 14.40 | 1.29 | 0.371 |
| hsa-miR-30d-5p | 5275 | 4068 | 4671 | 12.19 | 1.30 | 0.375 |
| hsa-miR-92a-3p | 44765 | 34115 | 39440 | 15.27 | 1.31 | 0.392 |
| hsa-mir-92a-2 | 20374 | 15260 | 17817 | 14.12 | 1.34 | 0.417 |
| hsa-let-7d-3p | 203 | 151 | 177 | 7.47 | 1.34 | 0.426 |
| hsa-mir-223 | 2572 | 1902 | 2237 | 11.13 | 1.35 | 0.435 |
| hsa-miR-223-3p | 2438 | 1776 | 2107 | 11.04 | 1.37 | 0.457 |
| hsa-mir-941-2 | 413 | 300 | 357 | 8.48 | 1.38 | 0.461 |
| hsa-mir-941-3 | 410 | 297 | 353 | 8.46 | 1.38 | 0.464 |
| hsa-mir-27a | 1705 | 1217 | 1461 | 10.51 | 1.40 | 0.487 |
| hsa-miR-128-3p | 205 | 146 | 175 | 7.45 | 1.40 | 0.487 |
| hsa-miR-941 | 836 | 596 | 716 | 9.48 | 1.40 | 0.489 |
| hsa-mir-128-1 | 127 | 90 | 109 | 6.76 | 1.41 | 0.492 |
| hsa-miR-106b-3p | 284 | 202 | 243 | 7.93 | 1.41 | 0.493 |
| hsa-mir-3184 | 4400 | 3114 | 3757 | 11.88 | 1.41 | 0.499 |
| hsa-mir-423 | 4400 | 3114 | 3757 | 11.88 | 1.41 | 0.499 |
| hsa-miR-1307-3p | 214 | 149 | 182 | 7.51 | 1.44 | 0.522 |
| hsa-miR-27a-3p | 1670 | 1133 | 1401 | 10.45 | 1.47 | 0.560 |
| hsa-miR-25-3p | 3398 | 2301 | 2849 | 11.48 | 1.48 | 0.563 |
| hsa-miR-486-3p | 551 | 368 | 459 | 8.84 | 1.50 | 0.582 |
| hsa-miR-486-5p | 551 | 368 | 459 | 8.84 | 1.50 | 0.582 |
| hsa-mir-486-2 | 550 | 367 | 459 | 8.84 | 1.50 | 0.583 |
| hsa-mir-941-4 | 427 | 275 | 351 | 8.46 | 1.55 | 0.633 |
| hsa-miR-3184-3p | 2731 | 1760 | 2245 | 11.13 | 1.55 | 0.633 |
| hsa-miR-423-5p | 2731 | 1760 | 2245 | 11.13 | 1.55 | 0.633 |
| hsa-miR-3615 | 261 | 165 | 213 | 7.74 | 1.58 | 0.660 |
| hsa-miR-222-3p | 1097 | 604 | 851 | 9.73 | 1.81 | 0.860 |
| hsa-mir-320b-2 | 214 | 113 | 163 | 7.35 | 1.90 | 0.925 |
| hsa-miR-320b | 443 | 228 | 336 | 8.39 | 1.94 | 0.957 |
| hsa-mir-320b-1 | 229 | 115 | 172 | 7.43 | 1.99 | 0.994 |
| hsa-mir-197 | 250 | 121 | 186 | 7.54 | 2.06 | 1.043 |
| hsa-miR-197-3p | 250 | 121 | 186 | 7.54 | 2.06 | 1.043 |
| a - Mac were prepared by overnight incubation of CD14-bead-isolated monocytes in the presence or absence of statin. Subsequently, cells were stimulated with LPS. After further 24 hours incubation, total RNA was isolated. Total RNA was isolated using the RNeasy Plus Mini kit. "Deep Sequencing" was performed by the "Core Unit DNA" (Universität Leipzig), using the "TrueSeqTMSmall RNA Sample prepkit 2" (illumina). The normalized data (counts) of LPS-stimulated Mac differentiated in the presence (Statin (A)) or absence (No statin (B)) of fluvastatin (10 µg/ml) are listed.  b - The mean of A and B was calculated and the 192 micro RNAs of the 4445 analyzed micro RNAs, which expressed a mean of >100 counts were included into the table. The Lg2_MeanA/B_ indicates the expression level of the various micro RNAs.  c - In order to define the down- or upregulation of the respective microRNAs, the expression of the micro RNAs in the presence of statin (A) was divided by the expression in the absence of statin (B).  d - The Lg2 of A/B was calculated and the table sorted by the Lg2_A/B_-value, ranging from the most down-regulated (negative values) to the most-upregulated values (positive values). | | | | | | |

**Supplemental References**

1. Loppnow H, Flad H-D, Dürrbaum I*, et al.* Detection of interleukin-1 with human dermal fibroblasts. *Immunobiology* 1989; 179:283 - 291.

2. Blum B, Herzbeck H, Loppnow H*, et al.* Antipeptide antibodies as probes for the biological activity of human interleukin-1. *Immunobiology* 1987; 175:309-310.

3. Herzbeck H, Blum B, Rönspeck W*, et al.* Functional and molecular characterization of a monoclonal antibody against the 165-186 peptide of human IL-1β. *Scand J Immunol* 1989; 30:549 - 562.
