# Supplementary material for: The differential statin effect on cytokine production of monocytes or macrophages is mediated by differential geranylgeranylation-dependent Rac1 activation
Source: Cell Death Dis. 2019 Nov 21;10(12):880. doi: 10.1038/s41419-019-2109-9 (PMC6872739; doi:10.1038/s41419-019-2109-9)
Supplement: Supplementary file 2 — supplemental legends [file 41419_2019_2109_MOESM2_ESM.docx]

**Supplement Figure 1A-C. Statins retain the cytokine production of monocyte-derived macrophages in the differentiation phase, but do not affect cytokine production of freshly-isolated monocytes**

(A) Statin-pretreatment retains the capacity of macrophages to produce IL‑6, TNF‑α and IL‑1ß. The figure shows a representative experiment, including TNF-data. This figure also emphasizes the lack of statin-effect in the unstimulated conditions (N), showing only background cytokine production. Mo and Mac (50000 cells/cm^2^; 6-well plate; 3 ml; statin, 10 µg/ml; LPS, 100 ng/ml) were prepared as described in Fig. 1A of the original manuscript. The IL‑6, IL‑1 and TNF in the supernatants was measured by ELISA. Four additional experiments with the same design were performed. Mean, SD and significances were calculated as described in Fig. 1C of the original manuscript (Mac vs. Mo, Mo‑Stat, or Mac‑Stat, respectively; i.e., black column vs. blue, orange or red column, respectively); *, p < 0.05; **, p < 0.01; ***, p < 0.001; ns, not significant). N, No LPS; L, LPS; M, No Statin; S, Statin. Legend below the figure: black letters - treatment at day 1; blue letters - treatment at day 2.

(B) The retainment effect is also visible at the mRNA-level. Mo and Mac (100000/cm^2^; 25 cm^2^-flasks) were prepared as described in Supplementary Fig. 1A (S1A). Total mRNA was isolated and quantitative PCR (”Omniscript- or iScript-protocol“) of various experiments for IL-6 (3, Omniscript-protocol; 3, iScript-protocol) and IL‑1ß (3, iScript-protocol) was performed. The ratio of GAPDH to IL‑6 or IL‑1ß, respectively, was calculated. The highest relative expression of each experiment was determined 100 % and the data of the mentioned experiments summarized. Statistics and color code as in S1A.

(C) Macrophages derived from “untouched” monocytes also show the retainment effect. Untouched monocytes (Mo) were prepared using the flow-through of the “Pan-kit” (Miltenyi), which binds T-cells, B-cells, NK-cells, dendritic cells and basophils of the MNC. Both fractions (“Untouched”, as well as “T, B, NK, DC, baso”) were treated like the standard CD14-bead-isolated monocytes (“CD14"; 50000 cells/cm^2^; 24-well plate; 1 ml; 10 µg/ml statin; 100 ng/ml LPS). Macrophages (Mac) were also prepared from all three preparations. IL‑1ß was measured in ELISA. This experiment was performed once. Please note the axis break. Statistics and color code as in S1A.

**Supplement Figure 1D - 1G. Statins retain the cytokine production of monocyte-derived macrophages in the differentiation phase, but do not affect cytokine production of freshly isolated monocytes**

(D) The retainment effect is not limited to fluvastatin. Mo and Mac (50000/cm^2^; 24-well plate), prepared as described in S1A, were incubated with 10 µg/ml of fluvastatin (Flu), simvastatin (Sim), atorvastatin (Ator; all from Calbiochem) or rosuvastatin (Rosu; Santa Cruz), and LPS-stimulated (100 ng/ml). The green arrow indicates the low IL‑1ß-level (i.e., 7.1 ± 0.7 pg/ml) in the supernatant of Mac differentiated in the absence of statin (None). One additional experiment with a similar result was performed. Statistics (None vs. statins) and color code as in S1A.

(E) The IL‑1-activity in the supernatants of the statin-pretreated macrophages can be attributed to activated (i.e., processed) IL‑1ß, whereas no IL‑1α-activity was present. Supernatants of Mo and Mac (100000 cells/cm^2^; 75 cm^2^-flask; 100 ng/ml LPS; 10 µg/ml fluvastatin) were analyzed in fibroblast assay, in the absence or presence of inhibitory antibodies to IL‑1α (0.1 µg/ml) or IL‑1ß (0.1 µg/ml), respectively (compare Supplement Table 1). The antibodies blocked 10 ng/ml of the recombinant IL‑1α and IL‑1ß to 17 and 5 %, respectively. Activity was calculated with respect to a recombinant IL‑1α-standard (10 U/ml equals 10 ng/ml). One additional experiment with a similar result was performed. Statistics (Without antibody vs. anti-IL‑1α or anti-IL‑1ß, respectively) and color code as in S1A.

(F) Differential expression of caspase-1 in the retainment. Cell lysates of Mac (cond 3 and 4) contain less caspase‑1 p10 than Mo (cond 1 and 2). Mac prepared in the presence of statin contained higher IL‑1ß-precursor levels (cond 4) than Mac prepared without statin (cond 3), however, no difference of caspase‑1 p10 expression in the Mac with or without statin was detectable. Cell lysates of Mo or Mac (200000 cells/cm^2^; 6-well plate; 3 ml; 20 µg/ml Stat; 100 ng/ml LPS) were used for Western blot of GAPDH, inactive caspase‑1 (p30), active caspase‑1 (p10) and IL‑1ß. The locations of the intracellular IL‑1ß-precursor (pre_IC_) and the intracellular mature IL‑1ß (mat_IC_) are indicated. The numbers at the right indicate the molecular weight (kDa) taken from a molecular weight standard. The IL‑1ß‑levels (pg/ml) in the supernatants (IL‑1ß_SN_) of the respective cultures, as measured in ELISA, are presented below the blot (red numbers). Two additional experiments with similar results were performed. Abbreviations and color code as in S1A. cond, condition.

(G) The retainment effect of the statin is initiated within 3 to 6 hours of the differentiation-phase. Mononuclear cells (MNC) were cultured in 24-well plates (50000 cells/cm^2^). The undifferentiated MNC (Mo) were incubated with LPS (100 ng/ml) in the absence (blue columns) or presence (orange columns) of fluvastatin (10 µg/ml). Supernatants were harvested after 24 hours. The differentiated MNC (Mac) were preincubated for 3, 6, 12 and 24 hours, respectively, in the absence (black columns) or presence (red columns) of fluvastatin (10 µg/ml). After these preincubation periods LPS was added to the Mac for additional 24 hours and the SN were harvested. Cytokines were measured in ELISA. The experiment was performed twice. Statistics (absence vs. presence of statin) and color code as in S1A.

**Supplement Figure 2. Rac1 is involved in the retainment of the cytokine production**

(A) Density analysis. Density analysis of the bands shown in manuscript Fig. 4A and three additional experiments was performed in “TotalLab” software and summarized. Arbitrary units (Rac1-GTP (arb U)) were calculated with respect to total Rac1 (Rac1). Statistics (comparisons are indicated by the lines) and color code as in S1A. Abbreviations as in Fig. 4A of the original manuscript.

(B) The NF‑kB-(p65)-activity in Mo and Mac is not affected by statin. Mo and Mac (100000/cm^2^; 75 cm^2^-flasks) were prepared as described in S1A. Incubation with LPS or without LPS (-) was 20 minutes. The nuclear fraction was isolated using “Nuclear Extract Kit” and NF‑kB-p65 measured in “NF‑kB Family Transcription Factor Assay Kit” (both Active Motif). -, unstimulated monocytes; N, no statin; S, statin added (10 µg/ml); G, GGPP (5 µM). Except the unstimulated monocytes (-) all monocytes and macrophages were stimulated with LPS (100 ng/ml) under the respective Mo or Mac protocol.

(C) Inhibitors of Rac1, PI3K and p38 block the retainment effect in a concentration-dependent manner. Mo and Mac (50000 cells/cm^2^) were cultured and stimulated with LPS (100 ng/ml) in 24-well plates, as described in S1A. To statin-treated macrophages (red columns; red plusses) the inhibitors of Rac1/TIAM1 (NSC23766; Tocris), PI3K (LY294002; Tocris) or p38 (SB203580; Invivogen) were applied in parallel cultures in various concentrations (grey columns; compare grayscale in the figure). IL‑1 and IL‑6-levels were measured in ELISA. One additional experiment with similar results was performed. Statistics ("Mac + Stat" vs. "Mac + Stat + Inhibitors"; other comparisons are indicated by the lines) and color code as in S1A.

(D) Rac1- and RhoA-activation are enhanced in parallel in statin-treated cells. Cells were incubated as described in Fig. 4A of the original manuscript. Pull down analysis with the respective Rac1 or RhoA “Activation Kits” (BK035 and BK036, respectively) was performed according to the manufacturer´s instructions. The number at the upper right part of each blot describes the increase of the band density in the statin-treated lane (density of “Statin” / density of “No statin”).

**Supplement Figure 3. microRNAs related to the NF‑kB pathway are involved in the retainment**

(A) PCR for miR-146a. Total RNA of Mac was isolated as described in the Methods. Reverse transcription was performed with the “TaqMan µRNA RT Kit”. Quantitative PCR was performed with the “TaqMan µRNA Assay”. Expression data were normalized to U6-expression and the expression of unstimulated (None) Mac was determined 0.100. Data of six independent experiments were summarized. Statistics as in S1A (comparisons are indicated by the lines).

(B) The NF‑kB-translocation-inhibitor DHMEQ blocks the IL‑1 production in the retainment. Mo (blue and orange) and Mac (black and red) were prepared as described in S1A (48-well plate; 50000 cells/cm^2^; 100 ng LPS/ml) in the absence (blue; black) or presence (orange; red) of fluvastatin (10 µg/ml). The cells were cultured in the absence (filled squares) or presence (filled circles) of various concentrations of the NF‑kB-translocation-inhibitor DHMEQ (Inh). The solvent DMSO (open triangles) was tested in parallel at the corresponding concentrations. Interleukin‑1 was measured in ELISA. One additional experiment with a similar result was performed.

**Supplement Figure 4. The retainment effect is paralleled by reciprocal expression of macrophage-related surface markers**

(A). The IL-10-expression in Mac prepared in the absence of statin is increased. Mo and Mac (25 cm^2^ flasks; 50000 cells/cm^2^; 100 ng/ml LPS; 10 µg/ml fluvastatin) were prepared as described in S1A. The supernatants of the cultures were harvested and analyzed in IL‑1ß-, IL‑6- and IL‑10-ELISA. The cytokine concentrations were normalized to the highest respective cytokine level (100 %). Four additional experiments with comparable results were performed. Statistics (Without vs. With statin) and color code as in S1A. In addition, the Mac were statistically different to Mac+Stat, Mo and Mo+Stat in all tested cytokines.

(B) CD163 is preferentially expressed in classical and intermediate Mo and Mac. Mo and Mac were prepared as described in S1A. In parallel, Mac were differentiated overnight in the presence of GM- or M-CSF, respectively. The CD163-expression in the CD14^+^/CD16^-^-, CD14^+^/CD16^+^-, CD14^-^/CD16^+^- or CD14^-^/CD16^-^-subpopulations was determined. The data show a representative experiment out of three.

(C) merTK is enhanced in Mac differentiated in the absence of statin and reduced in Mac differentiated in the presence of statin. Mo and Mac were prepared and cultured as described in S4B. The merTK-expression was measured in FACS-analysis and the fluorescence intensity plotted. The numbers in the green box reflect the mean fluorescence intensity (MFI) of the samples (derived from FlowJo). The grey line indicates the isotype control. Two additional experiments with similar results were performed. GM, GM-CSF (20 ng/ml; M, M-CSF (20 ng/ml); S, fluvastatin (10 µg/ml).

**Supplement Figure 5. The gating strategy used in FACS-analyses**

(A) Doublet exclusion. Cell clusters and doublets were excluded by gating the single cells in gate P1.

(B) Identification of monocytes. The monocytes to be investigated were identified in gate P2, excluding granulocytes, lymphocytes and cell debris.

(C) Exclusion of dead cells. The gate P3 includes the vital cells, which were not stained by 7‑AAD (or Zombie AquaTM in some experiments).

(D) Surface marker staining. The present example shows a double staining for CD14 and CD16. The four gates were determined using the respective isotype controls. They contain double negatives (Q1), CD14^+^/CD16^-^ (Q2), CD14^+^/CD16^+^ (Q3) and CD14^-^/CD16^+^ (Q4).

(E) Statin does not attenuate the viability of the tested cells. Viability of cells included in the FACS-analyses was measured by 7‑AAD-staining. The mean and SD of all seven experiments was calculated and plotted. Isol, freshly isolated monocytes. Statistical analysis was performed as described in S1A. ns, not significant. We also found that the statin did not alter the LDH-level in the supernatants (data not shown).
