# Supplementary figures and images for: The differential statin effect on cytokine production of monocytes or macrophages is mediated by differential geranylgeranylation-dependent Rac1 activation
Source: Cell Death Dis. 2019 Nov 21;10(12):880. doi: 10.1038/s41419-019-2109-9 (PMC6872739; doi:10.1038/s41419-019-2109-9)

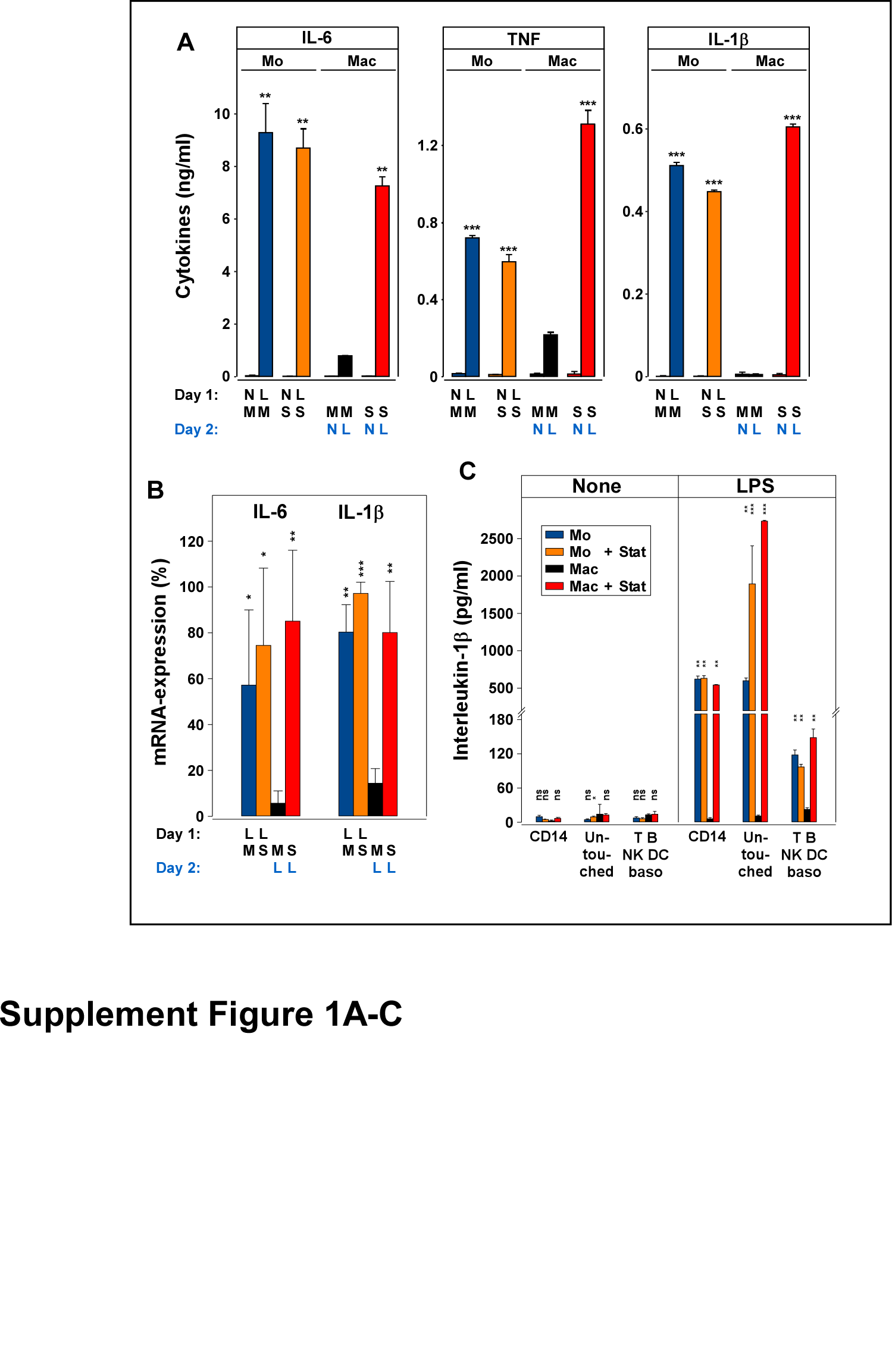

Supplement: Supplementary file 3 — supplemental Figure 1A-C [file 41419_2019_2109_MOESM3_ESM.tif]

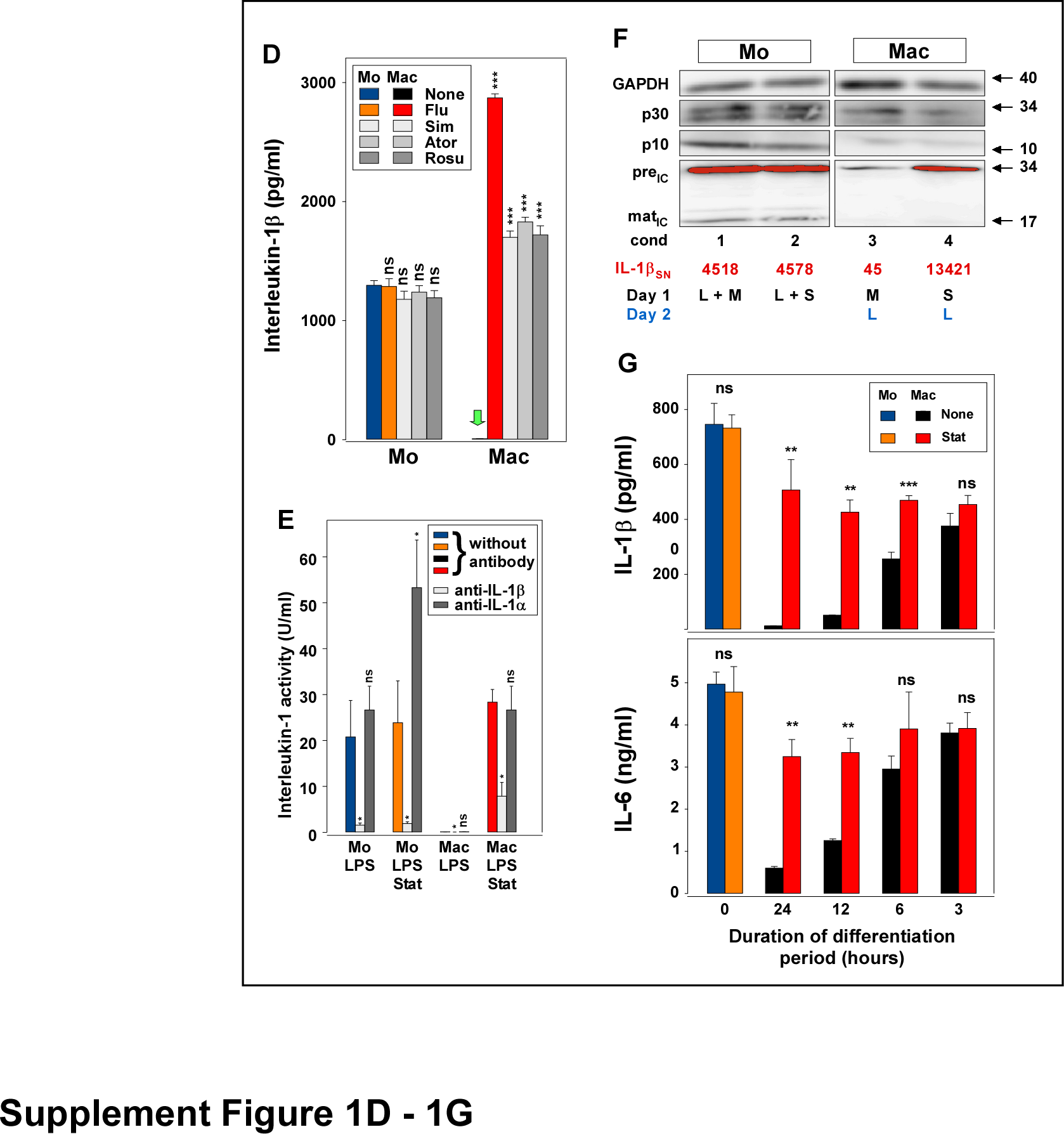

Supplement: Supplementary file 4 — supplemental Figure 1D-G [file 41419_2019_2109_MOESM4_ESM.tif]

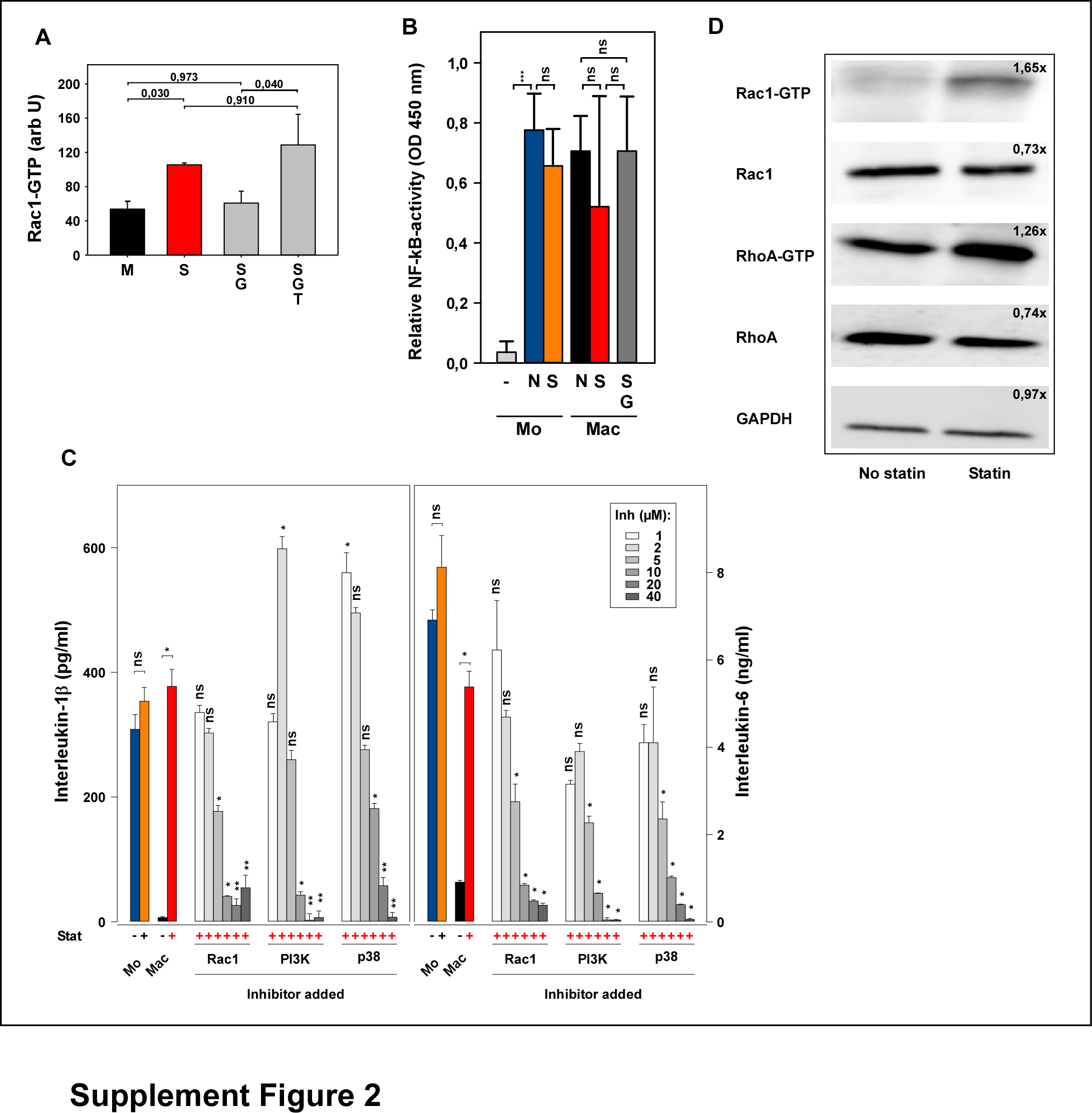

Supplement: Supplementary file 5 — supplemental Figure 2 [file 41419_2019_2109_MOESM5_ESM.tif]

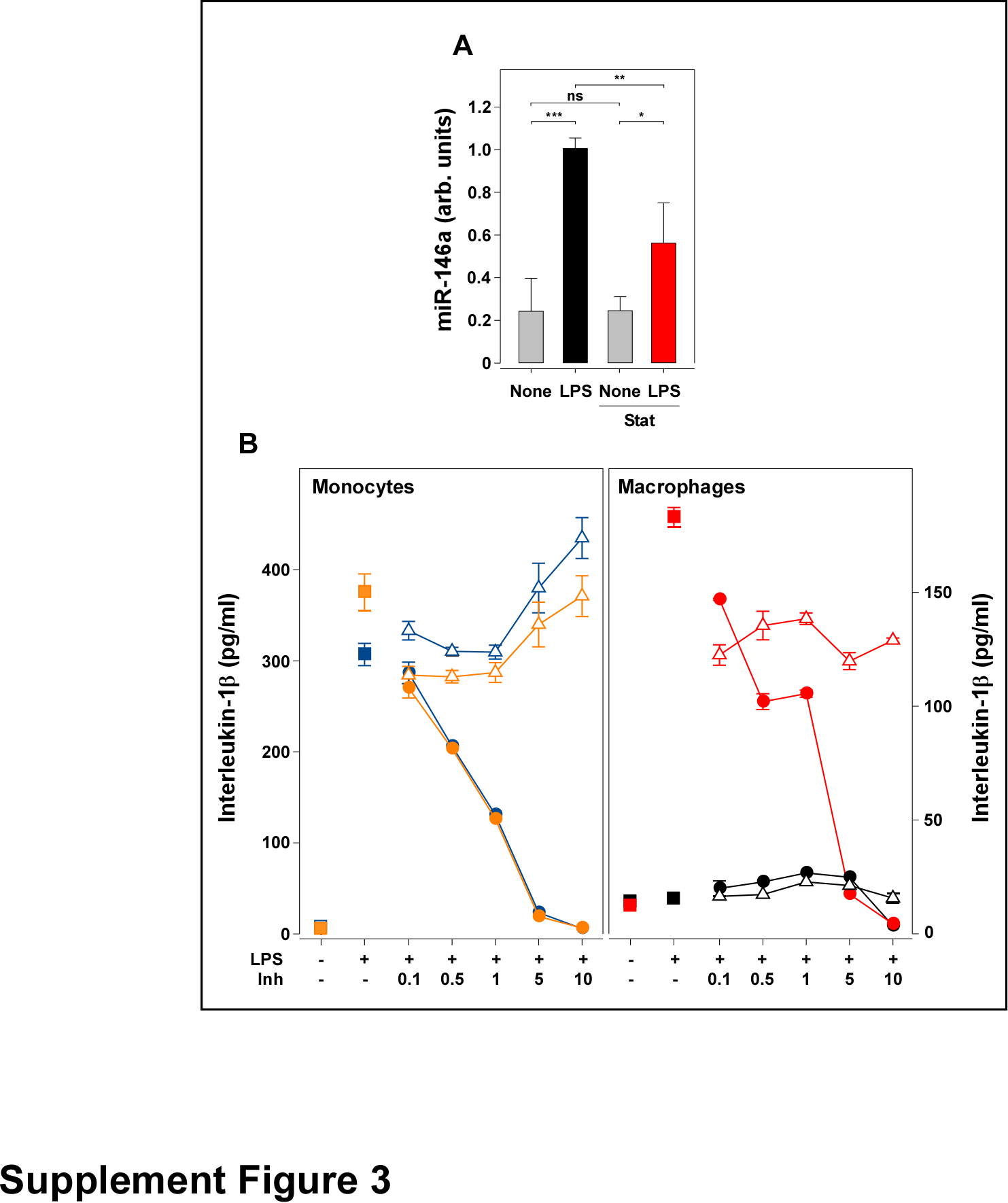

Supplement: Supplementary file 6 — supplemental Figure 3 [file 41419_2019_2109_MOESM6_ESM.tif]

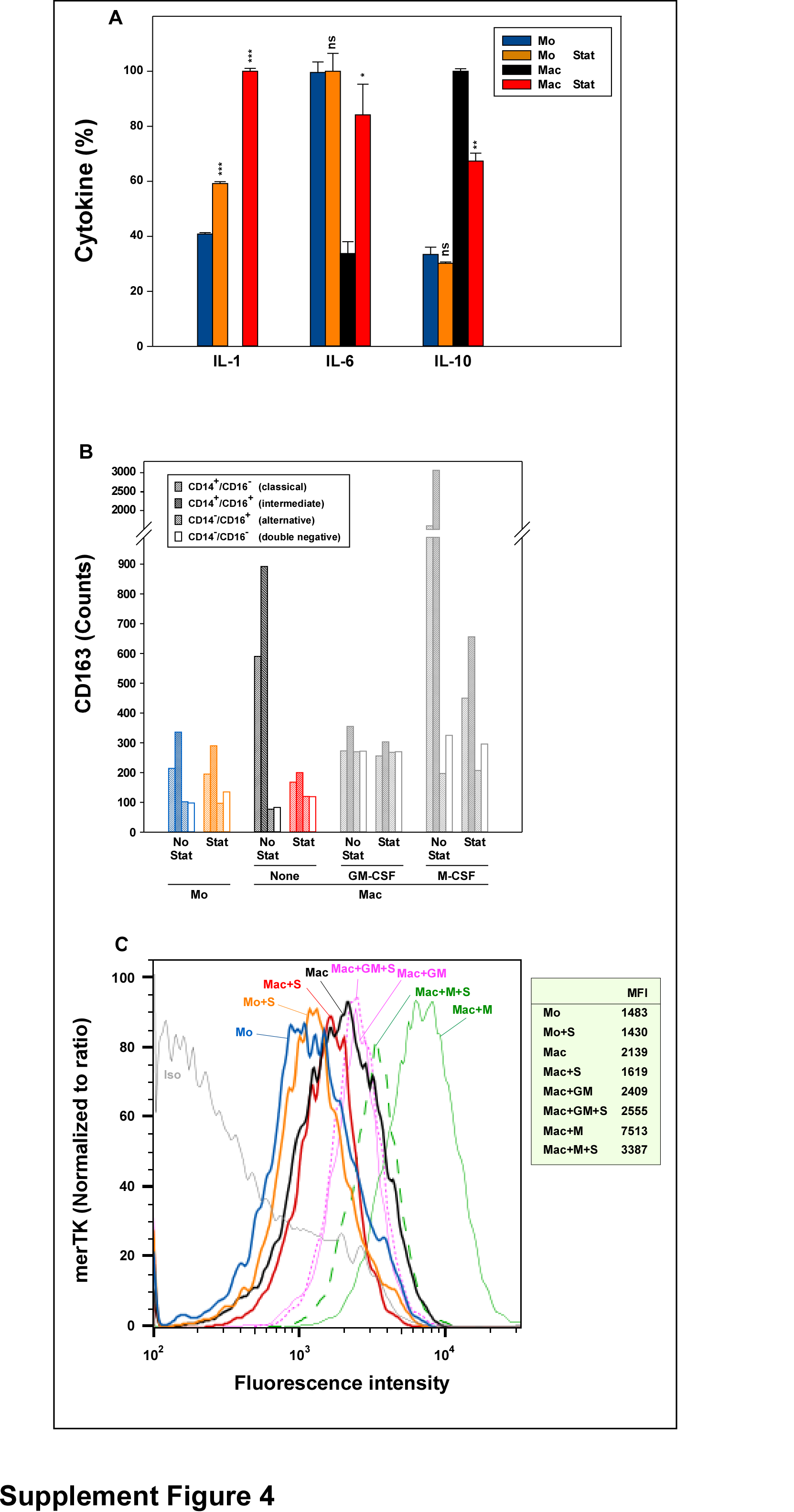

Supplement: Supplementary file 7 — supplemental Figure 4 [file 41419_2019_2109_MOESM7_ESM.tif]

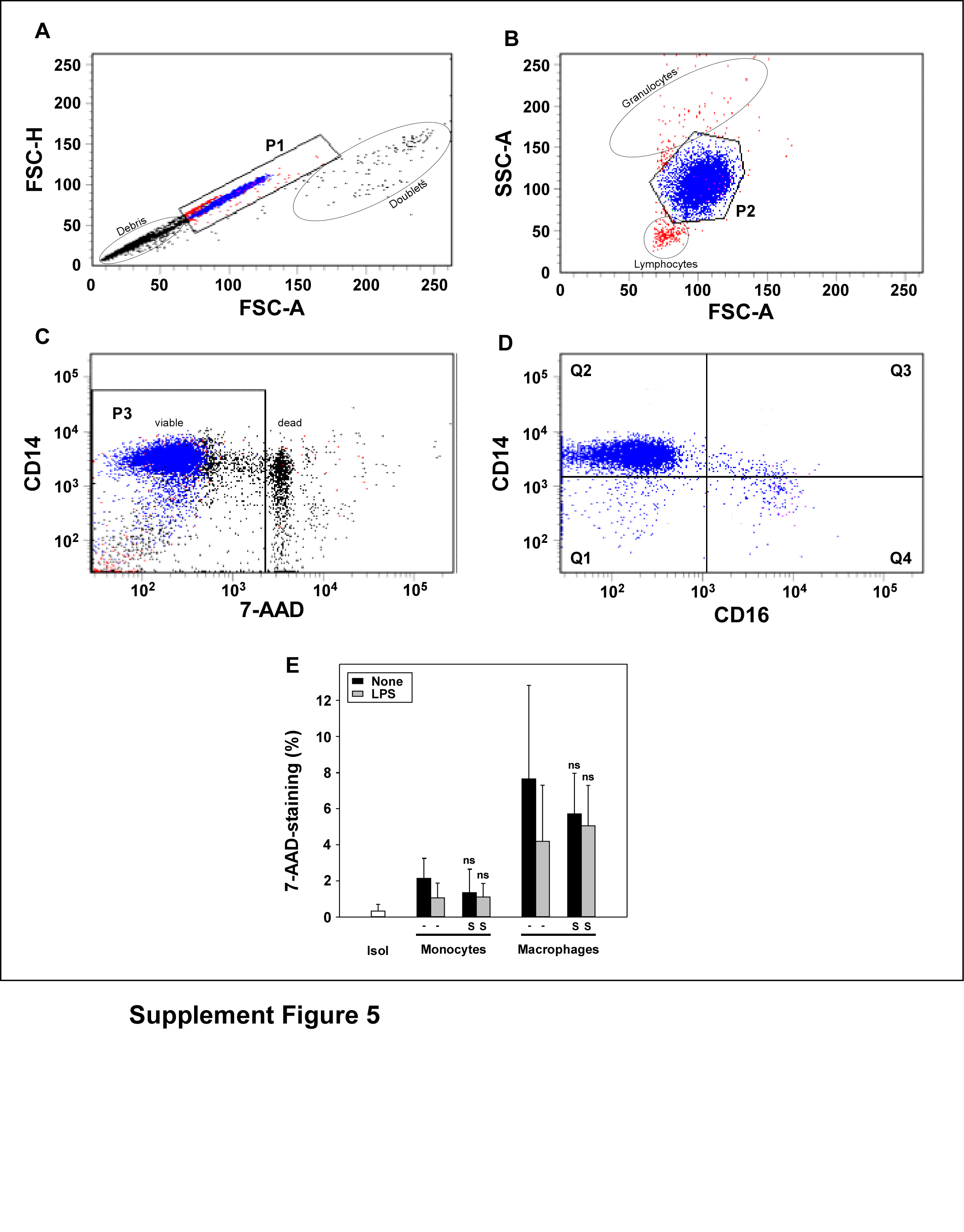

Supplement: Supplementary file 8 — supplemental Figur 5 [file 41419_2019_2109_MOESM8_ESM.tif]
